# Hypoxia-induced exosomal circPDK1 promotes pancreatic cancer glycolysis via c-myc activation by modulating miR-628-3p/BPTF axis and degrading BIN1

**DOI:** 10.1186/s13045-022-01348-7

**Published:** 2022-09-06

**Authors:** Jiewei Lin, Xinjing Wang, Shuyu Zhai, Minmin Shi, Chenghong Peng, Xiaxing Deng, Da Fu, Jiancheng Wang, Baiyong Shen

**Affiliations:** 1 Department of General Surgery, Pancreatic Disease Center, Ruijin Hospital, Shanghai Jiao Tong University School of Medicine, Shanghai, China; 2 Research Institute of Pancreatic Diseases, Shanghai Jiao Tong University School of Medicine, Shanghai, China; 3 State Key Laboratory of Oncogenes and Related Genes, Shanghai, China; 4 Institute of Translational Medicine, Shanghai Jiao Tong University, Shanghai, China

**Keywords:** Hypoxia, Exosomes, Pancreatic cancer, circRNA, ceRNA, Ubiquitination

## Abstract

**Background:**

circRNA has been established to play a pivotal role in tumorigenesis development in a variety of cancers; nevertheless, the biological functions and molecular mechanisms of hypoxia-induced exosomal circRNAs in pancreatic cancer remain largely unknown.

**Methods:**

Differentially expressed circRNAs in exosomes between hypoxic exosomes and normoxic exosomes in PC cells were verified by RNA sequencing. The expression of circPDK1 in PC tumors and PC patients was evaluated by qRT-PCR and ISH, and the biological functions of circPDK1 in PC were verified through a series of in vitro and in vivo experiments. Using Western blotting, Co-IP, RNA pull-down, ChIP, RIP, dual-luciferase assays, and rescue experiments, the underlying mechanism of circPDK1 was verified.

**Results:**

CircPDK1 was highly abundant in PC tumor tissues and serum exosomes and was associated with poor survival. Exosomal circPDK1 significantly promoted PC cell proliferation, migration, and glycolysis both in vitro and in vivo. Mechanistically, circPDK1 could be activated by HIF1A at the transcriptional level and sponges miR-628-3p to activate the BPTF/c-myc axis. In addition, circPDK1 serves as a scaffold that enhances the interaction between UBE2O and BIN1, inducing the UBE2O-mediated degradation of BIN1.

**Conclusions:**

We found that circPDK1 was activated by HIF1A at the transcriptional level by modulating the miR-628-3p/BPTF axis and degrading BIN1. Exosomal circPDK1 is a promising biomarker for PC diagnosis and prognosis and represents a potential therapeutic target for PC.

**Supplementary Information:**

The online version contains supplementary material available at 10.1186/s13045-022-01348-7.

## Introduction

Pancreatic cancer (PC) is a devastating digestive tract malignancy with delayed diagnosis, high rates of metastasis and occurrence, unfavorable prognosis, and insensitivity to chemotherapy [1, 2]. Notably, the five-year survival rate for patients with PC is less than 5% [3]. Despite the development of surgical technologies and chemotherapy treatments, the prognosis of PC remains disappointing. Hence, there is an urgent need to identify early diagnostic biomarkers, molecular mechanisms, and functional therapeutic targets for PC.

Hypoxia is widely acknowledged as one of the most important features of solid tumors and is involved in glycolysis, proliferation, and migration [4–6]. The activation of hypoxia-inducible factor 1α (HIF1A) is essential for solid tumors to adapt to hypoxic conditions and activate the transcription of various genes. The increase in HIF1A induces glycolysis, immune evasion, metastasis, and angiogenesis in hypoxic tumors [7, 8]. However, HIF1A protein becomes destabilized and gets degraded under aerobic conditions, implying that it cannot directly affect normoxic cancer cells.

Exosomes are released by various cells, including tumor cells, which are small single-membrane vesicles with diameters of 30–100 nm [9]. Exosomes typically carry various types of nucleic acids, including circRNAs, lncRNAs, and miRNAs, and play an important role in intercellular communication by transferring these contents [10]. Hypoxia may accelerate tumorigenesis by promoting exosomal release or by modulating exosome contents [5]. Exosomes derived from hypoxic tumor cells promote tumorigenesis through glycolysis, migration, invasion, and immune infiltration [6, 11]. Thus, hypoxia-induced exosomes can be a hypoxia signal carrier that directly affects normoxic cancer cells.

Circular RNAs (circRNAs) are a type of noncoding RNA formed by alternative splicing [12]. The loop structure of circRNAs makes them difficult to degrade in vivo, and they are widely abundant and specifically expressed in tumors and serum exosomes [13, 14]. Thus, circRNAs could be regarded as effective RNA biomarkers for the occurrence and development of tumors [15]. In terms of the mechanism, research demonstrates that circRNAs may modulate tumor progression by acting as ceRNA to sponge miRNA or functioning as scaffold [16].

The present study aimed to determine communication between hypoxic and normoxic PC cells modulated by hypoxia-induced exosomal circRNA and the underlying functions and mechanisms of circRNA derived from hypoxic exosomes during PC progression. We revealed that circPDK1 was significantly upregulated in exosomes from hypoxic cells compared with that in normoxic PC cells using RNA-seq. In addition, circPDK1 was highly abundant in PC tumors and serum exosomes and positively associated with a poor prognosis. Exosomal circPDK1 plays a role in PC by promoting cell viability, migration, and glycolysis in vitro and in vivo. In terms of its mechanism, circPDK1 activated c-myc-mediated glycolysis by sponging miR-628-3p, thereafter releasing BPTF. Functioning as a scaffold to strengthen the binding between UBE2O and BIN1, it enhances the effects of UBE2O on the ubiquitination and degradation of BIN1.

## Materials and methods

### Isolation, labeling, and quantification of exosomes

Pancreatic cell lines were incubated under normoxic conditions until they reached 70–80% confluence and then cultured in exosome-free medium under 20% (normoxia) or 1% (hypoxia) O_2_ for 48 h. Next, 20 mL of culture medium was harvested on ice and isolated by differential centrifugation [17]. The resulting sediment was resuspended with PBS. Exosome particle size and form, markers, and concentration were identified by transmission electron microscopy (TEM) (TECNAI 20; Philips, Netherland), Western blotting assay, and nanoparticle tracking analysis (NTA), respectively. Cells were collected for subsequent experiments after stimulation with 1 × 10^8^ exosomes for 48 h.

### Cell culture and transfection

All cells, including Aspc-1, Bxpc-3, CFPAC-1, MIA PaCa-2, PANC-1, PATU-8988, 293 T, and HPNE cells, were purchased from the Cell Bank of the Chinese Academy of Sciences (Shanghai, China). Aspc-1 and Bxpc-3 cells were cultured in RPMI-1640 medium, CFPAC-1 in IMDM, MIA PaCa-2, PANC-1, PATU-8988, HPNE, and 293 T cells in DMEM with 10% fetal bovine serum (FBS).

miR-628-3p mimics, miR-628-3p inhibitor, circPDK1 siRNA (si-circPDK1), UBE2O siRNA (si-UBE2O), HIF1A (si-HIF1A), circPDK1-WT, BPTF, circPDK1-MUT, circPDK1 truncations, BIN1 deletion mutations, and BIN1 and UBE2O were synthesized by Bioegene (Shanghai, China) and inserted into either lentivectors or plasmids. After transduction with lentivirus, 2 µg/mL puromycin was added to construct stable cell lines for 24 h. The miRNA mimics and siRNA sequences are listed in Additional file 1: Table S1, Additional file 2: Table S2. The circPDK1-WT and circPDK1-MUT sequences are listed in Additional file 3: Table S5. Lipofectamine 3000 was used as a transfection reagent for cell transfection.

### Cell proliferation assay

Cell viability was assessed using a CCK-8 assay (Dojindo, Japan). PC cells were harvested after treatment for 48 h, and 2 × 10^3^ PC cells were seeded in 96-well plates and incubated further. Then, 90 µL of completed medium with 10 µL of CCK-8 reagent was added and incubated for 2 h, after which the absorbance was measured at 450 nm. For the colony formation assay, 2 × 10^3^ PC cells were seeded in 6-well plates to evaluate PC cell proliferation ability. After 14 days, 1% crystal violet stain solution was used to fix the PC cells, and the number of colonies was counted. For the EdU assay, the EdU labeling kit (Epizyme, Shanghai, China) was used to assess cell proliferative ability. PC cells (3 × 10^4^) were seeded in 12-well plates for 48 h. The PC cells were then incubated with EdU reagent for 2 h, fixed with 4% paraformaldehyde, 0.5% Triton X-100, and followed by Hoechst staining. The EdU incorporation rate was defined as the proportion of EdU-positive cells (GREEN) to total Hoechst33342-positive cells (BLUE).

### Transwell migration assay

The migration ability of PC cells was assessed by transwell migration assay. Briefly, transwell chambers were plated with 6 × 10^4^ PC cells suspended in 200 µL serum-free medium and 700 µL complete medium in the lower chambers. After 24 h of culture, the upper chambers with migrated PC cells were fixed and stained with 1% crystal violet stain solution for 20 min, after which the migrated PC cells were counted manually by microscope. Relative proportions of migrated cells were calculated as the ratios (%) of treated to negative control cells.

### Western blotting, immunohistochemistry (IHC), immunofluorescence (IF), RNA fluorescence in situ hybridization (FISH), in situ hybridization (ISH), and immunoprecipitation (IP) assay

Cell proteins were boiled with RIPA buffer, loaded, and separated on 6%, 7.5%, and 10% SDS-PAGE gels, followed by their transfer onto polyvinylidene fluoride membranes and incubation with the indicated primary antibodies (Additional file 4: Table S3). β-actin and β-tubulin were used as controls. ECL reagents were used to detect the protein expression. All Western blotting reagents were purchased from EpiZyme (Shanghai, China). The IHC assay was performed using mouse xenograft tumor tissues and human PC tissues, as previously described [18]. IF assay was used to determine the subcellular localization of BIN1 and UBE2O in MIA PaCa-2 cells, as previously described [19]. Using FISH assay, the subcellular location of circPDK1 in MIA PaCa-2 cells was detected, as previously described [18]. Cy3-labeled circPDK1 probes were synthesized by RiboBio (Guangzhou, China). ISH assay was performed on the tissue microarrays to detect circPDK1 in PC tissues, as previously described [17], and the specific digoxin (DIG)-labeled probe of circPDK1 was designed by Servicebio (Wuhan, China). Protein–protein interactions were detected by performing the Co-IP assay, as previously described [19].

### Glycolysis analysis

The Glucose Uptake Assay Kit (abcam, USA), ATP Assay Kit (Beyotime, Shanghai, China), and Lactate Assay Kit-WST (Dojindo, Shanghai, China) were performed to assess the glycolysis level according to the manufacturer’s guidelines. Using the Seahorse XF96 Glycolysis Analyzer (Seahorse Bioscience, MA, USA), the ECAR and OCAR were measured according to the manufacturer’s guidelines.

### qRT-PCR, RT-PCR and subcellular fractionation assay

RNA was extracted using TRIzol reagent (Invitrogen, USA), and the HiScript III RT SuperMix (TOYOBO, Japan) was used for reverse transcription, followed by the AceQ Universal SYBR qPCR Master Mix (AG, China) to detect the RNA expression levels, and normalized to β-actin or U6. The divergent and convergent primers of circPDK1 and GAPDH were used to detect the circular characteristics of circPDK1 by RT-PCR. The primer sequences are listed in Additional file 5: Table S4. Using the PARIS Kit according to the manufacturer’s guidelines, RNA from subcellular fractionation was separated to assess the relative subcellular localization of circPDK1.

### Dual-luciferase reporter assay

Luciferase reporter vectors containing the 3ʹ-UTR binding site of circPDK1 and BPTF or the matched mutant sequence, c-myc-responsive transcriptional sequence, wild-type miR-628-3p transcriptional promoter or circPDK1 transcriptional promoter, and circPDK1 corresponding promoter mutant sequence were synthesized by Bioegene (Shanghai, China) and cloned into the reporter plasmids. Luciferase activity was assessed using a dual-luciferase reporter assay system (Synergy LX; BioTek, USA) and normalized to Renilla.

### Chromatin immunoprecipitation (ChIP)

The SimpleChIP Plus Sonication Chromatin IP Kit (CST, USA) was used to perform the ChIP assay according to the manufacturer’s instructions. Using 1% formaldehyde, MIA PaCa-2 cells were cross-linked for 10 min, followed by quenching with glycine. DNA fragments were sheared by sonication until they ranged from 200 to 500 bp. The nuclear lysate was immunoprecipitated with anti-HIF1A, anti-Pol II, or IgG antibodies. The purified DNA fragments were analyzed by qRT-PCR with specific primers; the ChIP primers are listed in Additional file 5: Table S4.

### RNA immunoprecipitation (RIP)

RIP experiments were performed using the Magna RNA-binding protein immunoprecipitation kit (Millipore, Bedford, MA, USA), according to the manufacturer’s instructions. The immunoprecipitated RNA in MIA PaCa-2 and 293 T cells was verified by qRT-PCR.

### RNA pull-down assay

The biotinylated RNAs were pulled down by incubating the MIA PaCa-2 cell lysates with streptavidin–agarose beads (Invitrogen, USA) according to the manufacturer’s instructions. The eluted proteins were identified by mass spectrometry analysis and Western blotting. circPDK1-Positive-probe-biotin (CTGGTGATTTTGCTTAATGTAGAT) and circPDK1-Negative-probe-biotin (GCAGTTATCTACATTAAGCAAAAT) were designed by RiboBio (Guangzhou, China). The mass spectrometry (MS) findings are provided as in Additional file 6: Table S8.

### Mice model assay

To evaluate the functions of exosomes in vivo, wild-type MIA PaCa-2 (5 × 10^6^ cells/0.1 mL PBS; *n* = 5 in every group) were injected subcutaneously on the right flank of 4-week-old male BALB/c nude mice. Once the subcutaneous tumor volume reached 100 mm^2^, the mice were randomly divided into four groups: 1 × 10^8^ exosomes/100 μL corresponding exosomes were injected into the tail vein every three days, and tumor volume was measured. After six injections, the mice were killed on the third day after the last injection. The subcutaneous tumors were weighed, fixed, and stained using IHC. To evaluate the migration of exosomes in vivo, a lung metastasis model was developed. Wild-type MIA PaCa-2 (1 × 10^6^ cells/0.1 mL PBS; *n* = 3 in every group) with indicated 1 × 10^8^ exosomes/100 μL exosomes were injected into the tail vein. The indicated exosomes were injected into the tail vein of the mice every seven days. After six injections, mice were killed on the third day after the last injection, and lung tissue was photographed and stained with hematoxylin and eosin (HE).

To evaluate the functions of circPDK1-WT and circPDK1-MUT in vivo, stably transfected MIA PaCa-2 cells (5 × 10^6^ cells/0.1 mL PBS; *n* = 5, every group) overexpressing circPDK1-WT or circPDK1-MUT were injected into the right flank of 4-week-old male nude mice. The subcutaneous tumor volume was calculated every 6 days, and the mice were killed on day 30. Subcutaneous tumors were weighed and subjected to immunohistochemistry (IHC). For the lung metastasis model, stably transfected MIA PaCa-2 cells (2 × 10^6^ cells/0.1 mL PBS; *n* = 3, every group) were injected into the tail vein of 6-week-old mice. The mice were killed on day 42, lung tissue was photographed, stained with HE, and lung metastasis nodes were counted.

### Atomic rotationally equivariant scorer (ARES)

BIN1 protein was predicted by the High-Performance Computing Center of Shanghai Jiao Tong University through artificial intelligence simulation and homology modeling. The 3D modeling of RNA and protein-RNA docking steps were consistent with previous reports [20–22].

### Statistical analysis

The means between different groups were analyzed by one-way analysis of variance, Student’s *t*-test, and Chi-square test. The statistical difference results were calculated using SPSS 20.0 and GraphPad Prism 7.0 and are shown as mean ± standard deviation (SD). Experiments were independently repeated at least three times. Statistical significance was set at P < 0.05.

## Results

### Exosomes derived from hypoxia PC cells promote the malignant biological behavior of PC cells in vitro

Hypoxia is an important characteristic of pancreatic cancer. Primary tumors with PO_2_ < 10 mmHg in patients were defined as hypoxic, since oxygen availability would decrease as the distance from tumor cells to the nearest blood vessels increases. This distance is 100–200 μm depending on the local oxygen concentration in the blood and oxygen consumption rates [23–25]. Cells cultured under 1% and 20% O_2_ conditions were defined as hypoxic and normoxic, respectively. Thus, PC cells cultured in different oxygen concentrations could be defined as hypoxic and normoxic, which might cause tumor heterogeneity among them, and the malignant potential of hypoxic PC cells might be higher (Additional file 7: Fig. S1A). To determine whether exosomes secreted from hypoxic PC cells could affect the malignant biological behavior of normoxic PC cells, exosomes derived from hypoxic PC cells and normoxic PC cells were identified by TEM and NTA (Additional file 7: Fig. S1B, C), which demonstrated typical round particles with a diameter of 100 nm. The results of Western blotting showed that the exosome markers TSG101, CD63, and CD81 were substantially upregulated in hypoxic exosomes (Additional file 7: Fig. S1D). These results indicated that hypoxia could increase exosome secretion in hypoxic PC cells, which was consistent with previous studies of other cancers [26, 27].

Next, we detected the malignant biological behavior of PC cells after treatment with normoxic exosomes or hypoxic exosomes by colony formation, EdU, CCK-8, and transwell assays. The results showed that compared with the PBS control or normoxic exosomes, exosomes derived from hypoxic PC cells considerably improved the proliferation and migration of MIA PaCa-2 and PANC-1 cells (Additional file 7: Fig. S1E–H). Western blotting also showed that E-cadherin was significantly inhibited after treatment with hypoxic exosomes compared to PC cells with normoxic exosomes. Conversely, N-cadherin and vimentin protein levels were increased (Additional file 7: Fig. S1I). This suggests that exosomes derived from hypoxic PC cells could promote the viability and migration of PC cells.

### Identification of circRNAs via RNA-seq in hypoxic PC cells exosomes

**Fig. 1:**
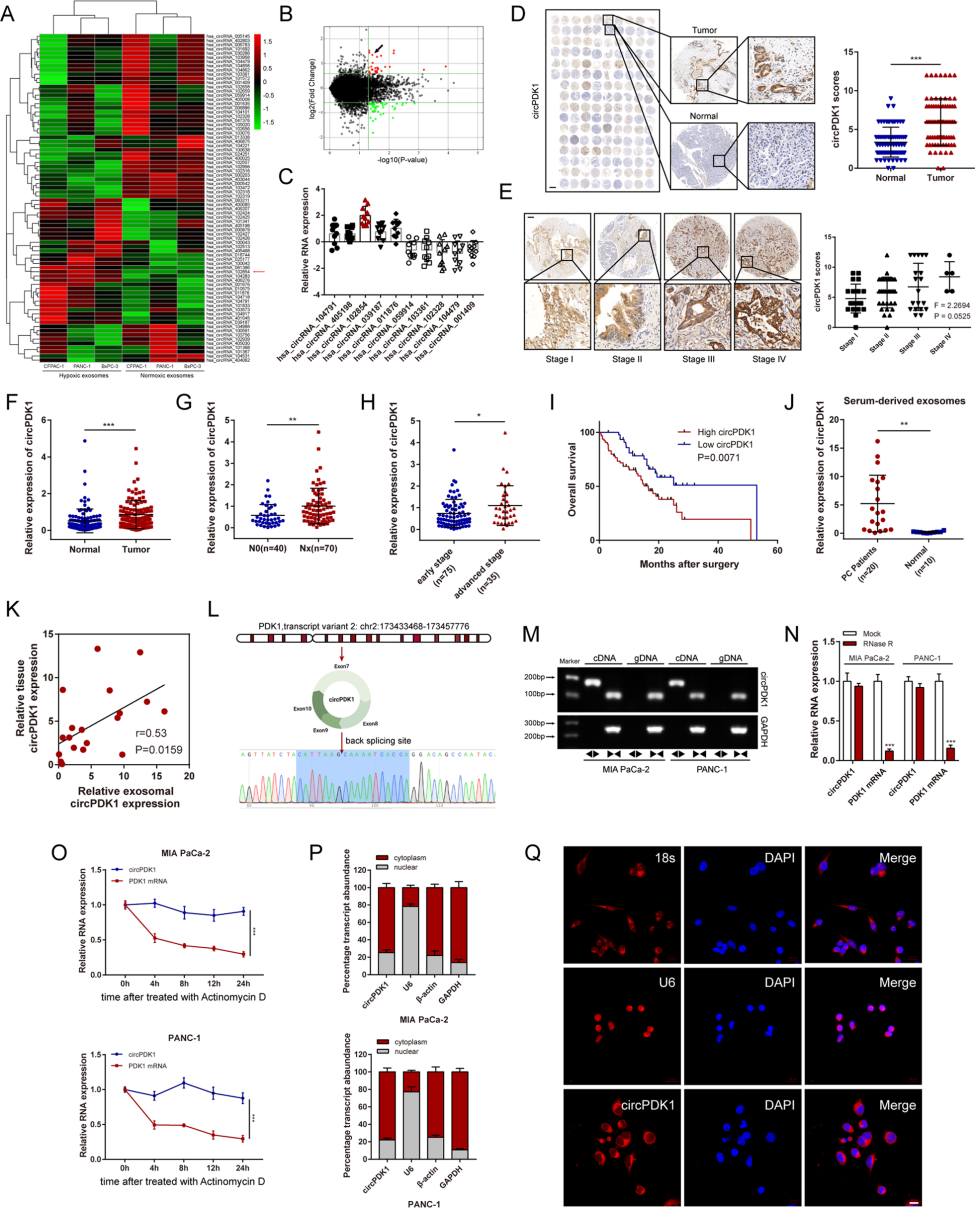
Identification of circPDK1 as an exosomal biomarker for PC and characteristic of circPDK1. **A** Heatmap showing the differentially exosomal circRNA expression between normoxic exosomes and hypoxic exosomes in PC cells. **B** Volcano plot displaying exosomal circRNAs that differentially notably between normoxic exosomes and hypoxic exosomes in PC cells. **C** Relative expression of top 5 most upregulated and downregulated circRNAs in 10 PC tumor and matched normal tissues. **D** The circPDK1 expression level in PC tumor tissues and matched normal tissues verified by ISH. Scale bar = 1000 μm. **E** The circPDK1 expression was detected in different TNM stage by ISH. Scale bar = 200 μm. **F** The expression of circPDK1 in 110 cases of PC tumor tissues and matched normal tissues. **G** The expression of circPDK1 in PC patients with lymph node metastasis. **H** circPDK1 expression in PC patients was divided by stage. **I** Prognostic analysis of circPDK1 in 110 cases of PC patients from our center. **J** circPDK1 in serum exosomes of PC patients (*n* = 20) and normal people (*n* = 10). **K** Correlation of circPDK1 expression between PC tumors and serum exosomes. **L** The back-splice junction site of circPDK1 was verified by Sanger sequencing. **M** The divergent primers for circPDK1 could be amplified by using PCR analysis, from cDNA but not gDNA, meanwhile, the divergent primers for GAPDH could not be amplified. **N** circPDK1 and PDK1 mRNAs expression after treatment with RNase R. **O** RNA abundance of circPDK1 and PDK1 after treatment with Actinomycin D. **P** Relative circPDK1 expression levels in subcellular fractions. **Q** Representative FISH images displaying the expression of circPDK1 in MIA PaCa-2 (RED). Scale bar = 20 μm. *P < 0.05; **P < 0.01; ***P < 0.001; ns, no significance

It has been widely confirmed that circRNAs are highly abundant in exosomes stably, which could be a key regulator of cancer [28]. To elucidate the role of hypoxia-induced exosomal circRNAs that may affect tumorigenesis, RNA sequencing was used to detect exosomes purified from normoxic and hypoxic PC cells. A total of 78 differentially expressed circRNAs were identified (FC (fold change) ≥ 1.5, *P* < 0.05) between hypoxic exosomes and normoxic exosomes (Fig. 1A, B).

### circPDK1 is highly expressed in PC

Next, the top five upregulated and downregulated circRNAs were analyzed by qRT-PCR in 10 cases of PC tumors and paired normal tissues. We discovered that circPDK1 was the most upregulated circRNA in PC (Fig. 1C). Then, an ISH assay in a tissue microarray with 75 pairs of tumor and matched normal tissues from PC patients was performed. The results revealed that circPDK1 was much more abundant in tumor tissues than in paired normal tissues (Fig. 1D), and the high expression of circPDK1 may be correlated with advanced pathological stage (Fig. 1E). Subsequently, 110 paired postoperative PC tissues and matched normal tissues from our center were detected using qRT-PCR to analyze the expression of circPDK1. The results demonstrated that circPDK1 was significantly upregulated in PC and was positively associated with lymph node metastasis, advanced pathological stage, and poor prognosis (Fig. 1F–I). To evaluate the clinicopathological and prognostic significance of circPDK1 in PC, clinical data were collected and analyzed in detail. We found that circPDK1 expression was significantly related to pathological stage (*P* = 0.019), T stage (*P* = 0.022), lymph node metastasis (*P* = 0.012), and distant metastasis (*P* = 0.034) (Additional file 8: Table S6). Furthermore, univariate analysis results demonstrated that high circPDK1 expression was an independent prognostic marker for patients with PC (Additional file 9: Table S7). CircRNA has been widely detected in serum exosomes and is considered a potential tumor marker in serum exosomes [17]. Thus, to explore whether circPDK1 could be detected in serum exosomes and its potential as a tumor marker, we collected blood samples from 20 patients with PC and 10 healthy individuals as controls. Interestingly, circPDK1 derived from serum exosomes was expressed abundantly in PC patients, while it almost did not exist in healthy controls (Fig. 1J). In addition, circPDK1 expression levels in serum exosomes were consistent with those in matched PC tumors (Fig. 1K), which made it possible to examine circPDK1 expression in serum samples. Taken together, these data reveal that circPDK1 is significantly upregulated in PC tumor tissues, which could be stably delivered and enriched in serum exosomes. Moreover, circPDK1 is barely expressed in serum exosomes and high circPDK1 expression is associated with poor prognosis in PC, which makes it a potentially promising circRNA biomarker for early diagnosis of PC.

### Characterization of circPDK1 in PC cells

circPDK1 (hsa_circRNA_102854, has_circ_0057104, chr2: 173433468–173457776) was generated from exons 7‒10 of PDK1 with a length of 401 nt. The back-splice site of circPDK1 was verified by Sanger sequencing (Fig. 1L). The divergent primers for circPDK1 could be amplified by PCR analysis, from cDNA but not gDNA; however, the divergent primers for GAPDH as a control could not be amplified (Fig. 1M). Compared with linear PDK1 mRNA, treatment with RNase R or actinomycin D demonstrated that circPDK1 was stabilized in PC cells (Fig. 1N, O). Subcellular fractionation and FISH assay results showed that circPDK1 was mainly localized in the cytoplasm (Fig. 1P, Q). Collectively, these results suggest that circPDK1 is an abundant, stably expressed, and cytoplasmic circRNA in PC.

### Exosomal circPDK1 is activated by HIF1A during hypoxic circumstance

**Fig. 2:**
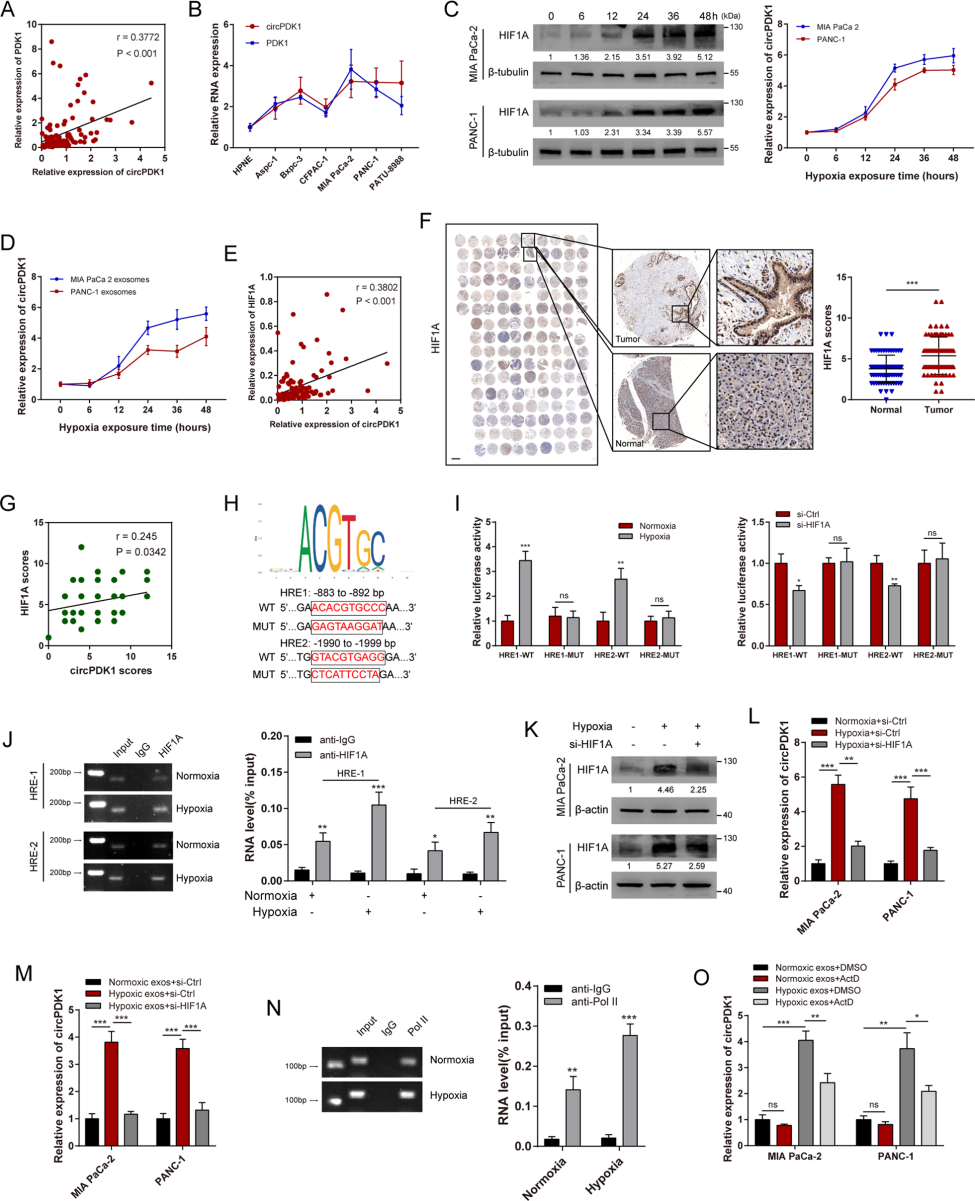
Exosomal circPDK1 is activated by HIF1A under hypoxic conditions. **A** The correlation between circPDK1 and PDK1 expression in 110 PC tissue samples. **B** The relative expression of circPDK1 and PDK1 mRNA in 7 PC cell lines. **C** The HIF1A protein level in PC cells exposed to different time under hypoxia (left) and circPDK1 expression level in PC cells exposed to indicated time in hypoxia (right). **D** The circPDK1 expression level in PC hypoxic exosomes exposed to indicated time in hypoxia. **E** The correlation between circPDK1 and HIF1A expression in 110 PC tissue samples. **F** The expression of HIF1A in PC tumor tissues and matched normal tissues verified by IHC assays in tissue microarrays. Scale bar = 1000 μm. **G** The correction of circPDK1 and HIF1A protein level was verified by IHC assays in tissue microarrays. **H** Upper schematic represents host gene PDK1 HREs obtained from JASPAR database. Dual-luciferase reporters were constructed with either of the two putative PDK1 HREs and matched mutant HREs in the PDK1 promoter region. **I** The luciferase intension of 293 T cells co-transfected with indicated luciferase reporter plasmids under hypoxia or loss of HIF1A. **J** ChIP assays were performed to assess the HREs interactions with the PDK1 promoter region. **K** The HIF1A protein levels in PC cells treated with indicated treatments. **L**, **M** The circPDK1 expression levels in PC cells treated with indicated treatments. **N** ChIP assays were performed to detect the binding strength between Pol II and the host gene PDK1 promoter in MIA PaCa-2 cells and normoxia and hypoxia. **O** The circPDK1 expression levels in MIA PaCa-2 cells treated with ActD (1 μg/mL) followed by indicated treatments. *P < 0.05; **P < 0.01; ***P < 0.001; ns, no significance

Studies have demonstrated that large amounts of circRNAs are transcriptionally activated by HIF1A under hypoxic conditions, based on exon-derived circRNAs, and their host linear genes would be generated from the same pre-mRNA [29, 30].Thus, we hypothesized that abundant circPDK1 in hypoxic exosomes may be due to the increased transcription of its host linear gene PDK1 activated by HIF1A. To verify our hypothesis, we first detected the co-expression relationship between PDK1 and circPDK1. The results revealed that circPDK1 expression level was positively correlated with its host linear gene PDK1 expression level (Fig. 2A). Moreover, compared with circPDK1 expression across the seven PC cell lines, circPDK1 expression was significantly non-differential (Fig. 2B). Next, we found that circPDK1 expression levels were notably overexpressed in both cells and exosomes during hypoxia in a time-dependent manner (Fig. 2C, D). Using qRT-PCR and IHC, we revealed that circPDK1 expression was positively associated with HIF1A expression, and HIF1A protein level was upregulated in PC (Fig. 2E–G). Using promoter sequence analysis tools (UCSC and JASPAR), two potential hypoxia-responsive elements (HREs) (ACACGTGCCC/GTACGTGAGG) were verified in the host gene of circPDK1 promoter regions (Fig. 2H). To determine whether HIF1A could directly interact with the two transcription start sites, a dual-luciferase reporter assay was performed in 293 T cells. The results confirmed that luciferase activity was enhanced in the reporter containing two individual wild-type HREs, while the reporter containing mutant HREs did not respond to hypoxia or HIF1A knockdown (Fig. 2I). Moreover, ChIP assays performed on the two predicted HREs upstream of the host gene for the circPDK1 promoter area suggested that HIF1A could directly bind to the PDK1 promoter region and enhance the transcription of host gene PDK1 under hypoxic conditions (Fig. 2J). Furthermore, the abundance of hypoxia-induced intracellular and exosomal circPDK1 was notably inhibited by knockdown of HIF1A (Fig. 2K–M). The ChIP assay results also verified that the binding between RNA polymerase II and the host gene PDK1 promoter site was enhanced under hypoxic conditions, which further verified that circPDK1 could be activated under hypoxia (Fig. 2N). In addition, the circPDK1 expression level in PC cells cultured with hypoxic exosomes was partly inhibited by treatment with ActD, and the circPDK1 stability in PC cells did not be affected under hypoxia (Fig. 2O and Additional file 10: Fig. S2A). Overall, these results indicate that the upregulation of circPDK1 in hypoxic exosomes is partly due to HIF1A activation.

### Hypoxia-derived exosomal circPDK1 promotes PC cell proliferation and metastasis in vitro and in vivo

To elucidate the role of hypoxia-derived exosomal circPDK1 in PC, exosomes with different abundances of circPDK1 derived from MIA PaCa-2 and PANC-1 cells were isolated and co-cultured with the corresponding donor cells, including four experimental groups: normoxic exosomes, hypoxic exosomes, hypoxic NC exosomes, and hypoxic sh1-circPDK1 exosomes. Using qRT-PCR, the abundance of circPDK1 in exosomes from each group was confirmed (Additional file 10: Fig. S2B). After treatment with the indicated exosomes, the abundance of circPDK1 in PC cells co-cultured with hypoxic sh1-circPDK1 exosomes was downregulated compared with that in the hypoxic NC exosomes group (Additional file 10: Fig. S2C).

**Fig. 3:**
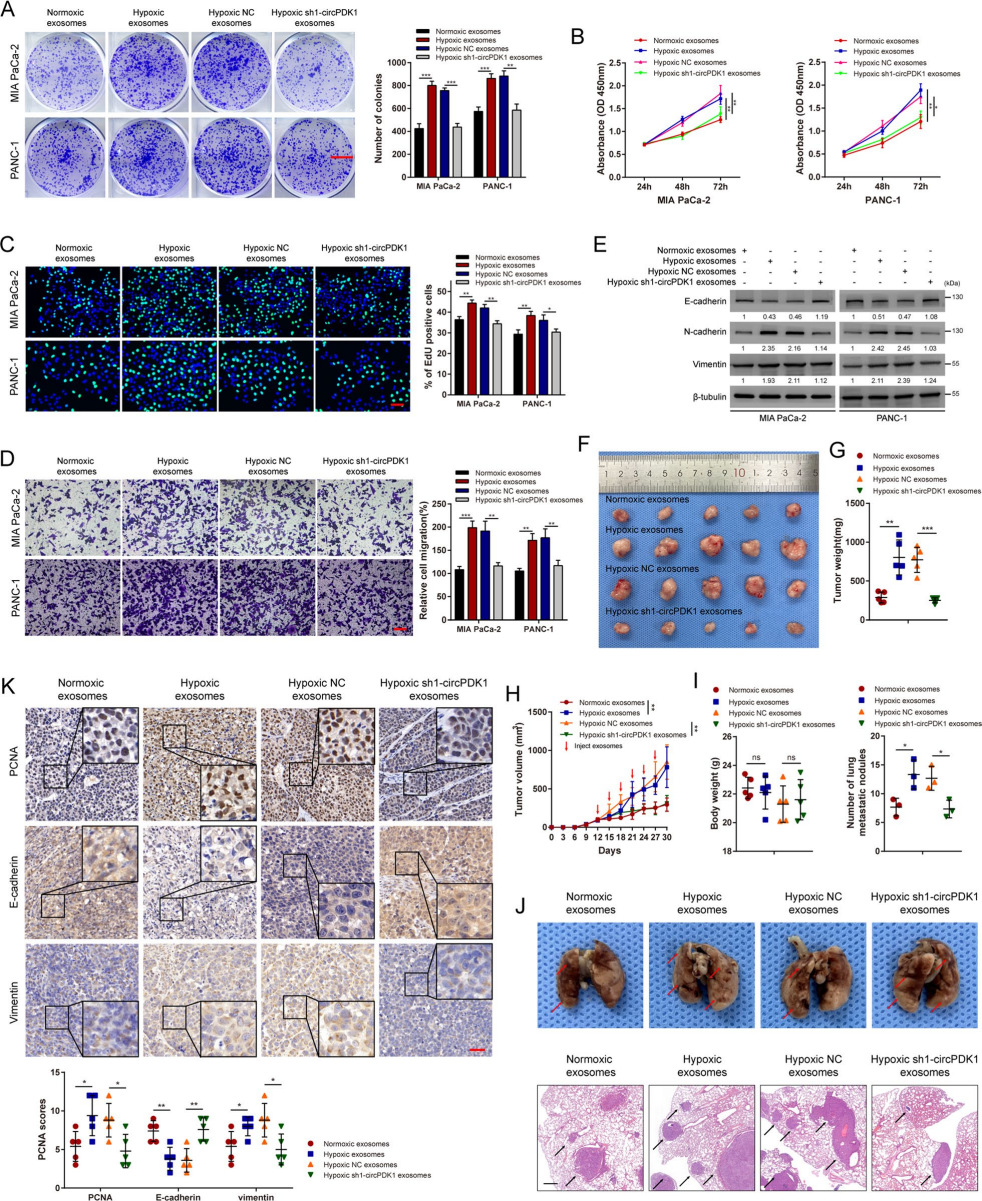
Hypoxia-derived exosomal circPDK1 promotes PC cells proliferation and metastasis in vitro and in vivo. **A** MIA PaCa-2 and PANC-1 cells were cultured in 6-well plates after treated with indicated exosomes. Scale bar = 1000 mm. **B** The viabilities of PC cells were detected by CCK-8 assays after treated with indicated exosomes. **C** The EdU assay was used to assess the cell proliferative potential of PC cells after treated with indicated exosomes. Scale bar = 50 μm. **D** Transwell migration assay of MIA PaCa-2 and PANC-1 after treatment with the indicated exosomes. Scale bar = 50 μm. **E** The expression of metastasis-related proteins was evaluated by Western blotting after treated with indicated exosomes. **F** Images of subcutaneous tumors. **G** The weight of subcutaneous tumors in each group. **H** Mice were injected through tail vein with indicated exosomes and the tumor volume was calculated every three days. **I** The body weight of mice in each group. **J** Representative photographs of the whole lung tissues and HE staining of lung metastatic nodules. **K** Representative photographs of PCNA, E-cadherin and vimentin IHC staining in subcutaneous tumors. Scale bar = 50 μm. *P < 0.05; **P < 0.01; ***P < 0.001; ns, no significance

Next, colony formation, EdU, CCK-8, and transwell assays were performed to detect the impact of hypoxia-induced exosomes on normoxic PC cell proliferation and migration in vitro. The results showed that PC cells treated with hypoxic exosomes showed enhanced viability and migration, whereas, after the elimination of circPDK1 from the hypoxic exosomes, this phenomenon of promoting proliferation and migration would disappear (Fig. 3A–D). In addition, Western blotting demonstrated that E-cadherin was significantly inhibited after treatment with hypoxic NC exosomes compared to PC cells with hypoxic sh1-circPDK1 exosomes. On the contrary, compared to treatment with hypoxic sh1-circPDK1 exosomes, N-cadherin and vimentin were notably upregulated in PC cells treated with hypoxic NC exosomes (Fig. 3E).

To explore the malignant effects of exosomal circPDK1 in vivo, a mouse xenograft tumor model (MIA PaCa-2 cell line) was established and intravenously injected with MIA PaCa-2 generated exosomes (normoxic exosomes, hypoxic exosomes, hypoxic NC exosomes, and hypoxic sh1-circPDK1 exosomes) once every three days. After six injections, the mice were killed on day 30 with the tumors harvested. As expected, hypoxic NC exosomes increased the volume and weight of xenograft tumors, while hypoxic sh1-circPDK1 exosomes abolished hypoxic exosomes to accelerate tumor growth (Fig. 3F–H). There was no significant difference in the body weights of nude mice among the groups (Fig. 3I). In the lung metastasis model, the number of lung metastatic nodules in the hypoxic sh1 exosome group was lower than that in the hypoxic NC group (Fig. 3J). To validate the functions of exosomal circPDK1 in mediating tumor growth and metastasis, xenograft tumor tissues of MIA PaCa-2 groups were stained with antibodies against PCNA, E-cadherin, and vimentin for IHC. The results showed that the expression of PCNA and vimentin was decreased in hypoxic sh1-circPDK1 exosome groups, whereas the expression of E-cadherin was increased in hypoxic sh1-circPDK1 exosome groups (Fig. 3K). These findings are consistent with the in vitro results showing that hypoxia-induced exosomal circPDK1 could also promote PC cell tumor growth and metastasis in vivo.

### circPDK1 functions as a miR-628-3p sponge in PC

**Fig. 4:**
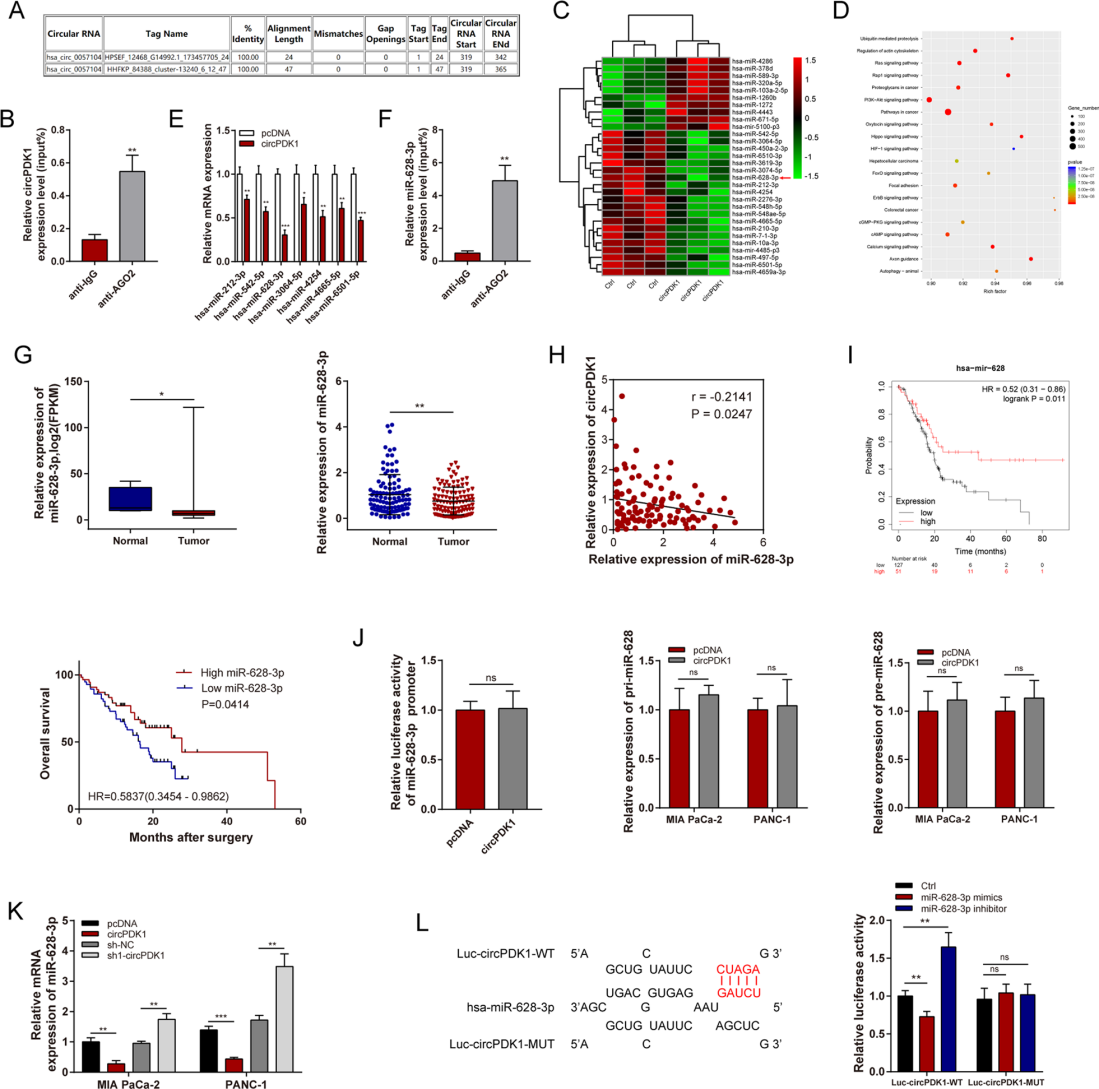
circPDK1 functions as a miR-628-3p sponge in PC. **A** The potential binding site between circPDK1 and AGO2 were predicted by circinteractome. **B** RIP assay was used to determine the relative expression of circPDK1 with rabbit AGO2 and IgG antibodies in MIA PaCa-2 cells. **C** Cluster heatmap showing the differentially expressed miRNAs between control and circPDK1 overexpression in MIA PaCa cells. **D** Pathway enrichment analysis were analyzed by using differentially expressed miRNAs after circPDK1 overexpression. **E** Seven potential target miRNAs expression in MIA PaCa-2 after circPDK1 overexpression. **F** RIP assay was used to determine the relative expression of miR-628-3p with rabbit AGO2 and IgG antibodies in MIA PaCa-2 cells. **G** The relative expression of miR-628-3p in PC patients were detected by the data from TCGA and our center. **H** The correction between circPDK1 and miR-628-3p were determined from our center. **I** Prognosis analysis of miR-628-3p were detected using survival data of PC patients from TCGA and our center. **J** Promoter luciferase activity in 293 T cells, pri-miR-628-3p and pre-miR-628-3p expression were verified after circPDK1 overexpression. **K** The expression of miR-628-3p were detected after circPDK1 overexpression and loss of circPDK1 in MIA PaCa-2 and PANC-1 cells. **L** Luciferase activity in 293-T cells co-transfected with Luc-circPDK1 wild-type or mutant sequence and miR-628-3p mimics or inhibitor. *P < 0.05; **P < 0.01; ***P < 0.001; ns, no significance

Many cytoplasmic circRNAs have been reported to function as ceRNAs by competitively sponging miRNAs [31], and given that circPDK1 is mainly localized in the cytoplasm, we assumed that cytoplasmic localization circPDK1 may act as a ceRNA by competitively sponging miRNAs. By researching the circinteractome online database (https://circinteractome.nia.nih.gov/index.html), we found that circPDK1 may bind to AGO2 (Fig. 4A). Thus, whether circPDK1 was enriched in AGO2-containing microribonucleoprotein complexes was verified by RIP assay (Fig. 4B). The findings revealed that circPDK1 may act as a miRNA sponge. Thus, we performed miRNA sequencing to screen potential differentially expressed miRNAs between the control groups and circPDK1 overexpression groups, which may be sequestered by circPDK1, and the efficiency of circPDK1 overexpression was also detected (Fig. 4C and Additional file 10: Fig. S2D). Pathway enrichment analysis suggested that differentially expressed miRNAs were enriched in ubiquitin-mediated proteolysis, PI3K-AKT signaling pathway, and HIF-1 signaling pathway, among others (Fig. 4D). Seven suppressed miRNAs were selected randomly, and then qRT-PCR was performed, and the expression levels of these miRNAs were confirmed in circPDK1 overexpressing MIA PaCa-2 cells. Among the seven candidates, miR-628-3p was shown to be significantly suppressed in circPDK1-overexpressing MIA PaCa-2 cells (Fig. 4E). RIP assays also demonstrated that miR-628-3p could bind to AGO2 (Fig. 4F). We found that miR-628-3p was notably suppressed in PC tumors in both TCGA data and our center, and inversely correlated with circPDK1 expression (Fig. 4H). Moreover, low miR-628-3p expression was associated with poor prognosis using clinical data from TCGA and our center (Fig. 4I). circRNAs can bind to RNA polymerase II directly, and the transcription of parental genes is activated [32]. To eliminate the possibility that circPDK1 modulates miRNA transcription, pri-miRNA, or pre-miRNA synthesis, we verified the impact of circPDK1 on the promoter regions, pri-miRNA, and pre-miRNA of miR-628-3p and confirmed that circPDK1 did not regulate promoter activity or pri-miRNA and pre-miRNA expression of miR-628-3p (Fig. 4J). miR-628-3p was downregulated after circPDK1 overexpression but upregulated in sh1-circPDK1 group in both MIA PaCa-2 and PANC-1 cells (Fig. 4K). Dual-luciferase reporter assays were performed, and the results showed that miR-628-3p directly binds to circPDK1 in 293 T cells (Fig. 4L). These findings indicate that circPDK1 may interact with miR-628-3p, thus functioning as a ceRNA sponge for miR-628-3p in PC cells.

### circPDK1 acts as a ceRNA to coordinate BPTF expression in PC

**Fig. 5:**
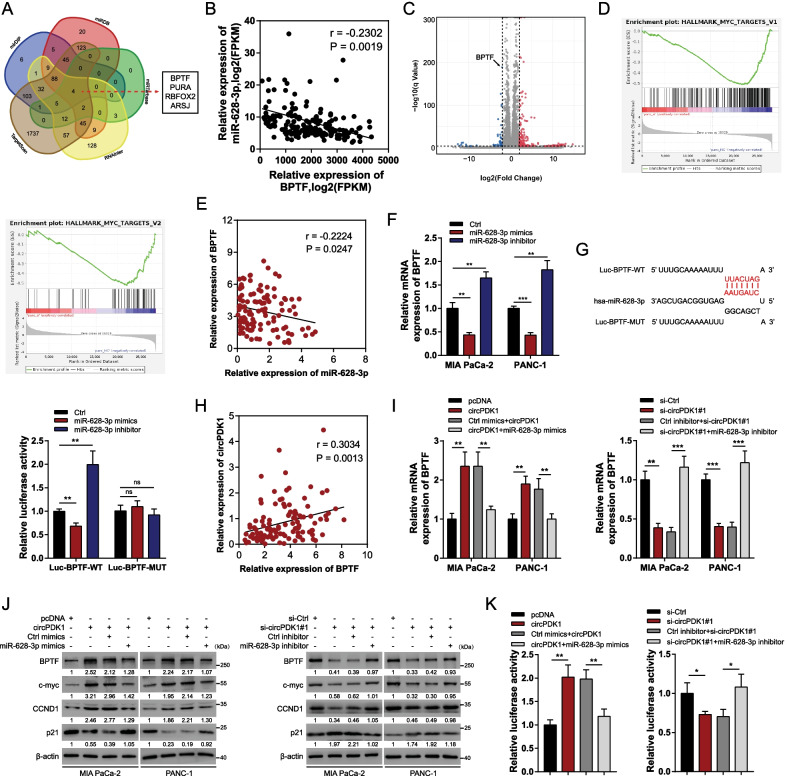
circPDK1 acts as a ceRNA to modulate BPTF expression in PC. **A** Potential target mRNAs of miR-628-3were predicted using mirDIP, RNAInter, miRDB, miRTarBase and TargetScan. **B** The correction between BPTF and miR-628-3p were determined from TCGA. **C** Volcano plot of differentially expressed protein-coding genes after the loss of circPDK1. **D** GSEA results were plotted to visualize the correlation between the expression of circPDK1 and c-myc relative pathway in PANC1 cells. **E** The correction between BPTF and miR-628-3p were determined from our center. **F** BPTF mRNA expression level was detected after treated with miR-628-3p mimics and inhibitor. **G** Luciferase activity in 293-T cells co-transfected with Luc-BPTF wild-type or mutant sequence and miR-628-3p mimics or inhibitor. **H** The correction between circPDK1 and BPTF were evaluated from our center. **I** BPTF expression was determined by qRT-PCR in MIA PaCa-2 and PANC-1 cells transfected with indicated treatments. **J** BPTF, c-myc, CCND1 and p21 expression was evaluated by Western blotting with indicated treatments. **K** Relative luciferase activity of Luc-BPTF with indicated treatment in 293 T cells. *P < 0.05; **P < 0.01; ***P < 0.001; ns, no significance

The underlying functions and mechanisms of action of miR-628-3p in PC remain unknown. Thus, potential targets of circPDK1-miR-628-3p axis were explored. Using the mirDIP, RNAInter, miRDB, miRTarBase, and TargetScan databases, BPTF, PURA, RBFOX2, and ARSJ were predicted to be potential downstream targets of miR-628-3p (Fig. 5A). We used data from TCGA to primarily detect a correlation between the expression of potential downstream targets and miR-628-3p. The results showed that the expression of BPTF was inversely associated with miR-628-3p, whereas that of PURA, RBFOX2, and ARSJ was not (Fig. 5B and Additional file 11: Fig. S3A–C). The RNA-sequencing findings after the loss of circPDK1 revealed 172 differentially expressed circRNAs (FC ≥ 2, adjusted *P* < 0.0001), including 135 upregulated and 37 downregulated protein-coding genes. BPTF ranked as one of the substantially downregulated protein-coding genes (Fig. 5C and Additional file 12: Fig. S4). We also identified a significant correlation between a c-myc pathway and circPDK1 using Gene Set Enrichment Analysis (GSEA; Fig. 5D). Notably, BPTF is required for c-myc transcriptional activity, and the BPTF-c-myc axis is involved in cell growth in pancreatic cancer [33]. Based on these results, we assumed that circPDK1 acts as a ceRNA to modulate the BPTF/c-myc axis by sponging miR-628-3p. To verify whether miR-628-3p could sponge BPTF directly, we further confirmed an inverse correlation between BPTF and miR-628-3p using data from our center (Fig. 5E). The mRNA expression levels of BPTF decreased after transfection with miR-628-3p mimics in both MIA PaCa-2 and PANC-1 cells, but increased after transfection with the miR-628-3p inhibitor (Fig. 5F). Dual-luciferase reporter assays showed that miR-628-3p could bind to the 3ʹ-UTR of BPTF (Fig. 5G). Therefore, we concluded that BPTF is a functional target gene of miR-628-3p. In addition, BPTF expression levels were positively correlated with circPDK1 expression levels in PC (Fig. 5H). Overexpressed circPDK1 substantially activated the BPTF-c-myc axis and modulated the c-myc downstream targets CCND1 and p21, whereas transfection with miR-628-3p mimics eliminated this effect. The inactivation of BPTF-c-myc axis and modulation of the two c-myc downstream targets induced by circPDK1 knockdown was also abolished by transfection with the miR-628-3p inhibitor (Fig. 5I, J). The same results were obtained using the dual-luciferase reporter assay (Fig. 5K). Functionally, miR-628-3p abolished the circPDK1 overexpression-induced promotion of cell proliferation and migration, similar to BPTF and miR-628-3p (Additional file 13: Fig. S5A–C). Collectively, circPDK1 serves as a ceRNA to bind to miR-628-3p from the sponging BPTF mRNA, thereby releasing BPTF from the inhibiting effects of miR-628-3p.

### circPDK1 interacts with BIN1 and enhances BIN1 ubiquitination

**Fig. 6:**
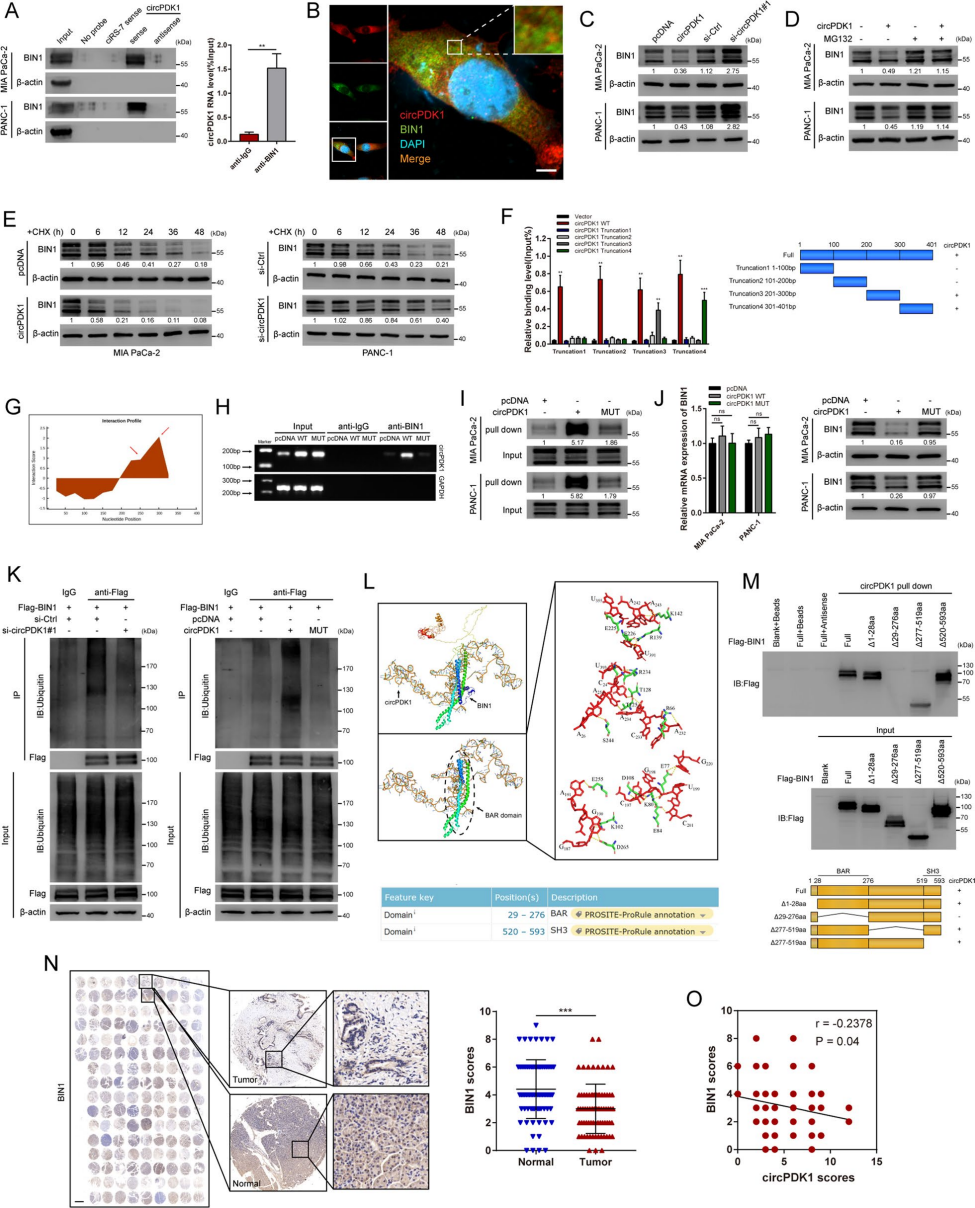
circPDK1 interacts with BIN1 and enhances BIN1 ubiquitination. **A** RNA pull-down verified the interaction of circPDK1 with BIN1. β-actin was used as a negative control. RIP assay was used to determine the relative expression of circPDK1 with rabbit BIN1 and IgG antibodies in MIA PaCa-2 cells. **B** Co-localization of circPDK1 (RED) with BIN1 proteins (GREEN), respectively, in MIA PaCa cells. Scale bar = 5 μm. **C** The expression of BIN1 in protein level after circPDK1 overexpression or loss of circPDK1. **D** The BIN1 protein level in MIA PaCa-2 and PANC-1 cells with circPDK1 overexpression treated with MG132 (20 μM) for 12 h. **E** The BIN1 protein level in indicated time point after treated with cycloheximide (CHX, 10 µg/mL) in transfected PC cells. **F** The interaction of circPDK1 truncations with BIN1 was confirmed by RIP assay in 293 T cells (left) and schematic diagram of circPDK1 full-length and truncations (right). **G** Interaction profile between circPDK1 and BIN1 was predicted by catRAPID. **H** RIP assays were performed using anti-BIN1 antibodies in MIA PaCa-2 cells by RT-PCR. **I** RNA pull-down assay was used to determine the interaction between circPDK1-WT or circPDK1-MUT and BIN1. **J** BIN1 mRNA and protein level were detected after transfected with circPDK1-WT or circPDK1-MUT. **K** IP assays verified the ubiquitination modification level of BIN1 in MIA PaCa-2 cells with indicated treatments. **L** The specific binding sites between circPDK1 and BIN1 was accurately construct by Atomic Rotationally Equivariant Scorer (ARES) (top) and the protein domain of BIN1 queried from UniProt (bottom). **M** RNA pull-down assays was performed to verify the interaction between truncated BIN1 protein and circPDK1 (top) and schematic of truncated BIN1 protein (bottom). **N** The expression of BIN1 in PC tumor tissues and matched normal tissues verified by IHC assays in tissue microarrays. Scale bar = 1000 μm. **O** The correction of circPDK1 and BIN1 protein level was verified by IHC assays in tissue microarrays. *P < 0.05; **P < 0.01; ***P < 0.001; ns, no significance

We investigated whether circPDK1 exerts its functions by interacting with RNA-binding proteins using RNA pull-down assays and silver staining. We found that abundant 200, 120, 75, 65, and 35 kDa proteins were associated with circPDK1 (Additional file 20: Fig. S12A). Mass spectrometry revealed that 65 kDa Myc box-dependent-interacting protein 1 (BIN1) was one of the topmost likely RNA-binding proteins of circPDK1 and that it might be abundant among circPDK1-associated proteins. Thus, we assumed that BIN1 would interact with circPDK1 directly; this hypothesis was next confirmed by RNA pull-down and immunoprecipitation assays, while a control circRNA named ciRS-7 [34], which acts as a miRNAs sponge, would not bind with BIN1 (Fig. 6A). Moreover, FISH assays demonstrated that circPDK1 was co-localized with BIN1 in the cytoplasm (Fig. 6B and Additional file 14: Fig. S6A). Although circPDK1 did not regulate BIN1 mRNA expression level, the BIN1 protein levels were significantly regulated after circPDK1 was altered (Additional file 15: Fig. S7A and Fig. 6C). In addition, circPDK1 expression induced reduced BIN1 protein levels that could be restored by proteasome inhibitor (MG132) (Fig. 6D). After treatment with cycloheximide (CHX), BIN1 had a shorter half-life in PC cells overexpressing circPDK1, while it had a longer half-life in PC cells that downregulated circPDK1 (Fig. 6E). To investigate the circPDK1 regions that interact with BIN1, 293 T cells were co-transfected with various truncations of circPDK1, follow RIP assays, truncation 3 and 4 interacted with BIN1 (Fig. 6F). Furthermore, those truncations were degraded after treatment with RNase R, which demonstrated that those truncations were linear (Additional file 16: Fig. S8). Based on the bioinformatic online database catRAPID, two predicted potential binding sites in truncations 3 and 4 were mutated (Fig. 6G). Using Sanger sequencing, mutations and the back-splice site in circPDK1-MUT were detected, and the results revealed that circPDK1-MUT was circularized correctly and possessed the same back-splice site with circPDK1-WT (Additional file 17: Fig. S9). Moreover, mutations in circPDK1 reduced the interaction between circPDK1 and BIN1 (Fig. 6H, I). This indicated that circPDK1-WT interacted with BIN1 in truncations 3 and 4, but not circPDK1-MUT in PC cells. circPDK1-MUT did not affect BIN1 at either the mRNA or protein level (Fig. 6J). Therefore, we assumed that circPDK1 might destabilize BIN1 proteins by interacting with it. Suppression of circPDK1 significantly inhibited the ubiquitination levels of BIN1, while circPDK1 overexpression notably enhanced the ubiquitination levels of BIN1, but this effect was eliminated by mutation (Fig. 6K). Furthermore, the atomic rotationally equivariant scorer (ARES) was used to accurately construct the 3D structure of the BIN1 regions that interact with circPDK1 computationally. The analysis results showed that circPDK1 could bind to BIN1 only in the BAR domain region, which was consistent with catRAPID (Fig. 6L). To confirm this prediction, four deletion mutants of BIN1 were designed, followed by an RNA pull-down assay, and the results showed that deletion of the 29‒276 aa region (BAR domain) of BIN1 abolished its interaction with circPDK1 (Fig. 6M). IHC assay also revealed that BIN1 protein levels were downregulated in PC tissues and negatively correlated with circPDK1 in tissue microarray (Fig. 6N, O). Together, these data indicate that circPDK1 might modulate the stability of BIN1 by interacting with the BAR domain region of BIN1, thus promoting its ubiquitin-dependent degradation.

### circPDK1 acts as a scaffold to enhance the binding of BIN1 proteins with UBE2O

**Fig. 7:**
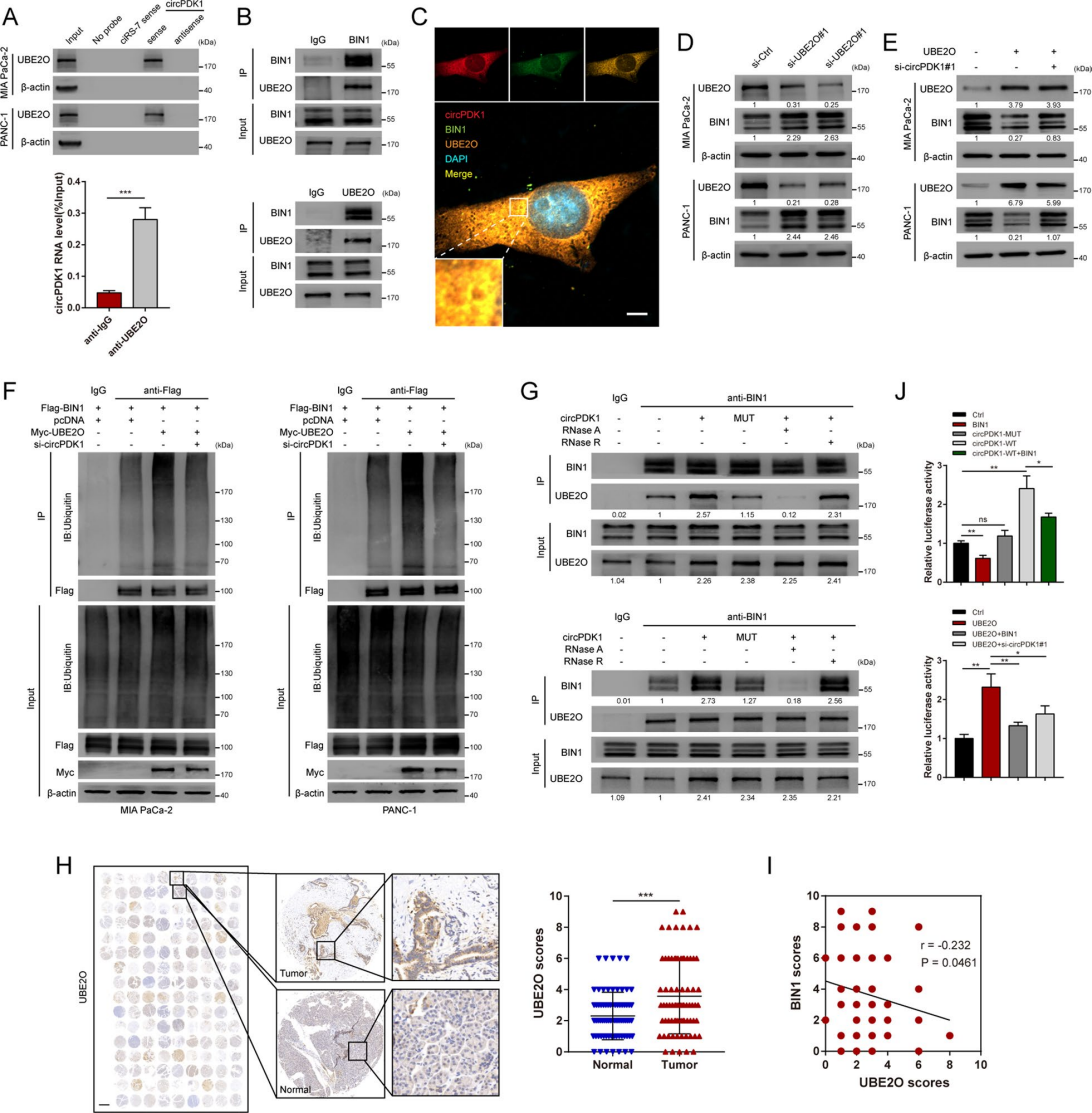
circPDK1 acts as a scaffold to enhance the binding of BIN1 proteins with UBE2O. **A** RNA pull-down verified the interaction of circPDK1 with BIN1. β-actin was used as a negative control. RIP assay was used to determine the relative expression of circPDK1 with rabbit UBE2O and IgG antibodies in MIA PaCa-2 cells. **B** Co-IP assays demonstrated the binding of UBE2O with BIN1 in MIA PaCa-2 cells. **C** Co-localization of circPDK1 (red), BIN1 proteins (green), and UBE2O proteins (orange) in MIA PaCa cells. Scale bar = 5 μm. **D** UBE2O and BIN1 protein levels were detected after knockdown of UBE2O. **E** UBE2O and BIN1 protein levels were detected with indicated treatments. **F** Ubiquitination modification levels of BIN1 in MIA PaCa-2 and PANC-1 cells with UBE2O overexpression and loss of circPDK1. **G** The interaction between UBE2O and BIN1 proteins were verified by Co-IP assays in MIA PaCa-2 cells. RNase A (10 μg/ml) and RNase R (100 U/ml). **H** The expression of UBE2O in PC tumor tissues and matched normal tissues verified by IHC assays in tissue microarrays. **I** The correction of UBE2O and BIN1 protein level was verified by IHC assays in tissue microarrays. Scale bar = 1000 μm. **J** The luciferase activity of c-myc responsive transcriptional reporter was evaluated with indicated treatments. *P < 0.05; **P < 0.01; ***P < 0.001; ns, no significance

To further screen the ubiquitin-enzyme-mediated BIN1 ubiquitination, mass spectrometry was performed after RNA pull-down. Interestingly, only a ubiquitin conjugating enzyme named UBE2O, which displays both E2 ubiquitin-conjugating enzyme and E3 ubiquitin ligase activities [35], was found among all participants. To verify whether UBE2O participates in the ubiquitination effects of circPDK1 on BIN1, RNA pull-down and RIP were performed, and the interaction between circPDK1 and UBE2O was confirmed, while ciRS-7 would not bind to UBE2O (Fig. 7A). Co-immunoprecipitation (Co-IP) analysis was performed, and results indicated that UBE2O bound to BIN1 (Fig. 7B). Furthermore, FISH and IF assays indicated the colocalization of circPDK1, UBE2O, and BIN1 mainly in the cytoplasm (Fig. 7C and Additional file 14: Fig. S6B). Similarly, the qRT-PCR results demonstrated that the RNA levels of BIN1 and circPDK1 were not affected by UBE2O, but BIN1 protein levels were notably increased after UBE2O downregulation (Additional file 15: Fig. S7B, C and Fig. 7D). Next, we investigated whether circPDK1 is necessary for the ubiquitin ligase activity of UBE2O for BIN1. The results demonstrated that the loss of circPDK1 notably weakened the effects of UBE2O on the ubiquitination and degradation of BIN1 (Fig. 7E, F). Furthermore, Co-IP was analyzed to verify whether circPDK1 could function as a scaffold to enhance the interaction between UBE2O and BIN1. The results showed that the interaction between UBE2O and BIN1 would be enhanced in MIA PaCa-2 PC cells transfected with circPDK1-WT, but not transfected with circPDK1-MUT. In addition, the associations between UBE2O and BIN1 were severely destroyed when treated with RNase A, but not treated with RNase R (Fig. 7G). Because circRNAs were resistant to RNase R, they would be degraded by RNase A. IHC assay also indicated that UBE2O protein levels were increased in PC tissues and negatively correlated with BIN1 (Fig. 7H, I). Collectively, circPDK1 could serve as a scaffold to enhance the binding of UBE2O and BIN1, thus facilitating the effects of UBE2O on the ubiquitination and degradation of BIN1.

### BIN1 is a functional downstream mediator of circPDK1 and UBE2O

Functionally, compared with control or circPDK1-MUT, circPDK1-WT significantly promoted PC cell proliferation and migration both in vitro and in vivo. In addition, circPDK1-MUT promoted the proliferation and migration of PC cells. Moreover, circPDK1-WT overexpression-induced PC cell proliferation and migration could be eliminated by BIN1 overexpression in vitro (Additional file 18: Fig. S10A–C, Additional file 19: S11A–D). IHC assays showed that PCNA and vimentin were upregulated in circPDK1-overexpressing mice subcutaneous tumors, whereas E-cadherin was downregulated (Additional file 19: Fig. S11E). Compared with the control, UBE2O accelerated PC cell proliferation and migration, which could be reversed by BIN1 overexpression and loss of circPDK1 (Additional file 20: Fig. S12B–D). These findings indicate that circPDK1 plays an oncogenic role partially by acting as a scaffold between UBE2O and BIN1 to enhance the degradation of BIN1.

### circPDK1 activates c-myc through two non-interfering axes

Recent evidence has demonstrated that BIN1 is a tumor suppressor that interacts with c-myc, thereby limiting c-myc transcriptional activity [36, 37]. To verify whether UBE2O and circPDK1 are involved in BIN1-induced limited c-myc transcriptional activity in PC, a c-myc-responsive transcriptional luciferase reporter was transfected into MIA PaCa-2 PC cells with the indicated treatment. The c-myc-responsive transcriptional reporter luciferase activity was notably increased after transfection with circPDK1-WT, but not transfected with circPDK1-MUT, and this increase was partly abolished by BIN1. The luciferase activity increased by UBE2O was eliminated by BIN1 overexpression and loss of circPDK1 (Fig. 7J). Taken together, circPDK1 and UBE2O could activate c-myc transcriptional activity by degrading the BIN1 protein. To explore whether there are any cross-activation axes between the two axes, we detected the effect of BIN1 and UBE2O on the expression of miR-628-3p and BPTF, and vice versa. The results demonstrated that BIN1 and UBE2O did not affect miR-628-3p or BPTF expression (Additional file 21: Fig. S13A–C). At the same time, miR-628-3p or BPTF could not regulate BIN1 or UBE2O at the RNA or protein level (Additional file 21: Fig. S13D–E), suggesting that circPDK1 could activate c-myc through two no cross-talk axes.

### circPDK1 promotes aerobic glycolysis via c-myc activation

**Fig. 8:**
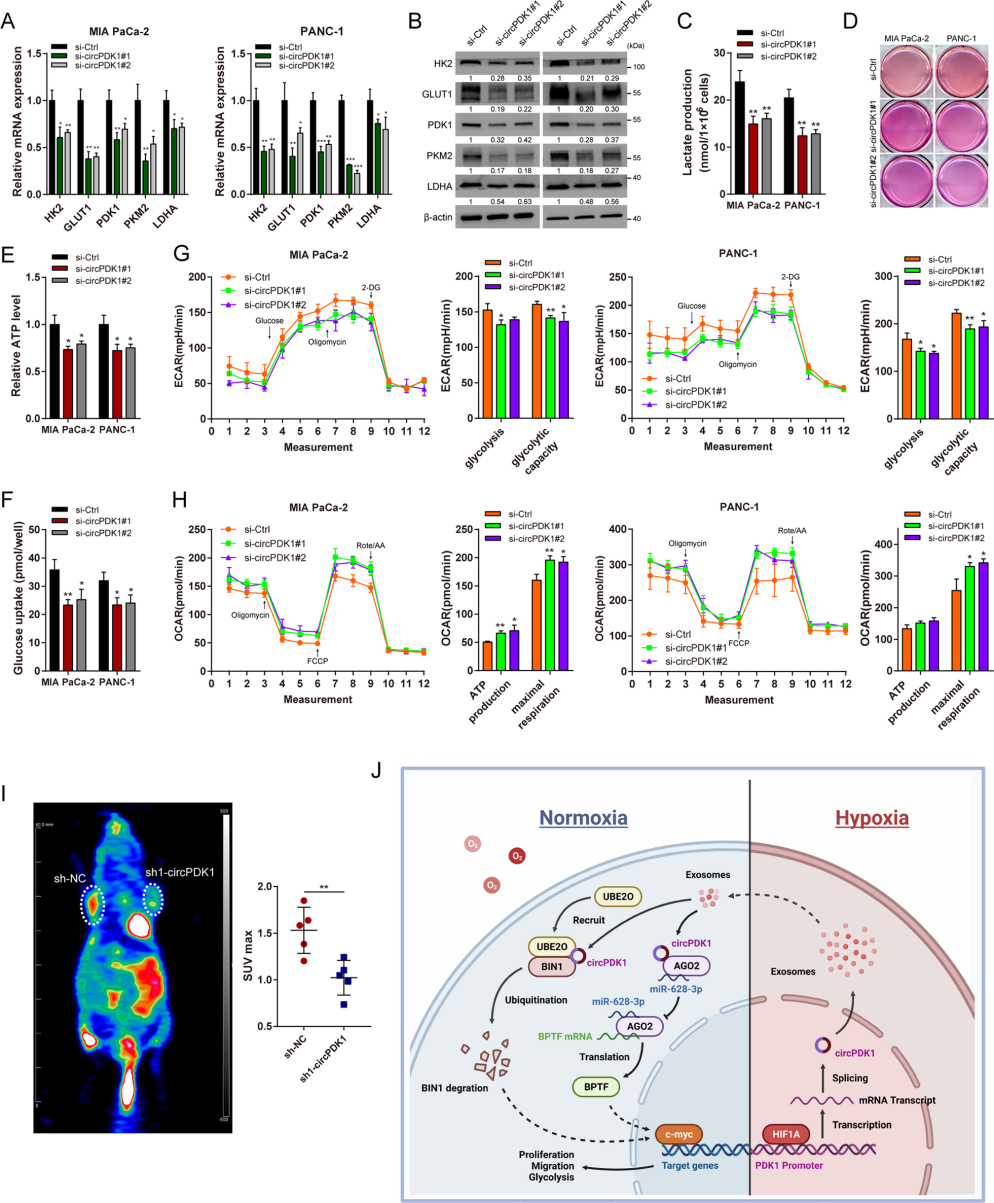
circPDK1 promotes aerobic glycolysis via c-myc activation. The glycolytic genes expressions were evaluated by **A** qRT-PCR and **B** Western blotting after loss of circPDK1. **C**, **D** Lactate production, **E** ATP production, **F** glucose uptake, **G** OCAR and **H** ECAR assays were performed to measure the glycolysis level in PC cells after loss of circPDK1. **I** PET-CT was performed to assess the glucose metabolism in mice subcutaneous tumors. **J** Proposed model demonstrating that hypoxia-induced exosomal circPDK1 promotes PC tumorigenesis via c-myc activation by modulating miR-628-3p/BPTF axis and degrading BIN1. *P < 0.05; **P < 0.01; ***P < 0.001

As a common target of two axes mediated by circPDK1, c-myc is known to be a key mediator of the Warburg effect by directly activating various target glycolytic genes to meet the demands of rapid proliferation and metastasis [38]. Thus, we explored whether circPDK1 is associated with aerobic glycolysis. qRT-PCR and Western blotting were performed to analyze the glycolytic genes, and the results demonstrated that loss of circPDK1 notably inhibited the expression of these glycolytic genes (Fig. 8A, B). Lactate production, ATP production, and glucose uptake could be reduced by loss of circPDK1 (Fig. 8C–F), as well as the extracellular acidification rate (ECAR) and oxygen consumption rate (OCR) (Fig. 8G, H). In vivo, PET-CT scanning indicated that the loss of circPDK1 would significantly inhibited glucose metabolism in mice subcutaneous tumors (Fig. 8I). Furthermore, IHC assays indicated that PDK1, LDHA, and c-myc were upregulated in circPDK1-WT-overexpressing the subcutaneous tumor (Additional file 19: Fig. S11E). To verify whether circPDK1 could promote glycolysis by activating c-myc through two axes, rescue experiments were performed. Functionally, BIN1 reversed the circPDK1-WT overexpression induced the promotion of glycolysis (Additional file 18: Fig. S10D–F), similar to that of miR-628-3p (Additional file 13: Fig. S5D–F). In addition, circPDK1-MUT promoted the enhancement of glycolysis in PC cells. Meanwhile, loss of circPDK1 and increased BIN1 would also abolish the UBE2O induced potentiation in glycolysis (Additional file 20: Fig. S12E–G). Collectively, these data indicate that c-myc mediates the functions of circPDK1 in glycolysis.

## Discussion

PC remains a serious threat among the digestive tract malignancy to human health due to its high metastasis, low surgical rates, high recurrence risk, and chemotherapy resistance. Although surgical technologies and comprehensive gemcitabine-based chemotherapeutics have been developed, the benefits to PC survival rates remain small [1]. Hypoxia is widely acknowledged as a significant characteristic of rapidly growing solid tumors and has an important impact on glycolysis, growth, and migration [6]. Hence, hypoxia levels will result in tumor heterogeneity among solid tumor cells, and present a higher malignant potential in hypoxic cells. In this study, we found that hypoxia stimulates the release of exosomes and the concentration of exosomes, which was consistent with previous studies in other cancers [26, 27]. In addition, circRNAs are stably abundant in exosomes and play an important role in tumor progression. This study aimed to determine communication between hypoxic and normoxic PC cells modulated by hypoxia-induced exosomal circRNA and the underlying functions and mechanisms of circRNA derived from hypoxic exosomes on the progression of PC.

Using RNA-seq analysis, we identified that circPDK1 was notably upregulated in exosomes from the medium supernatant of hypoxic PC cells. The expression level of circPDK1 in PC was detected, revealing that circPDK1 was markedly upregulated in PC tissues and serum exosomes, which was related to a poor prognosis. Notably, circPDK1 expression in serum exosomes was positively correlated with that in corresponding PC tumors, and there were few circPDK1 in healthy individuals. This revealed that circPDK1 in serum exosomes was stably secreted by PC tumor tissue, which may support a novel diagnostic biomarker for early PC patients.

Functionally, exosomal circPDK1 may significantly promote PC growth, metastasis, and glycolysis both in vitro and in vivo, indicating a tumor-promoting role in PC. Abundant circRNAs are transcriptionally activated by HIF1A under hypoxic conditions, based on exon-derived circRNAs, and their host linear genes would be generated from the same pre-mRNA; besides, circRNA might be modulated by HIF1A through activating transcription of the circRNA host gene [29, 30, 39]. In fact, the host gene of circPDK1, PDK1 is overexpressed in PC and transcriptionally activated by HIF1A, and parts of specific interactive regions have been verified [40–43]. Here, we discussed the transcriptional factor HIF1A that upregulates circPDK1 expression under hypoxia by interacting with the HREs of its host gene PDK1 promoter and investigated its specific binding regions.

Previous research has indicated that the molecular mechanism of circRNAs could be dependent on the subcellular localization of the circRNAs, with cytoplasm-localized circRNAs potentially acting as ceRNAs by sponging miRNAs [31]. We found that circPDK1 was mainly abundant in the cytoplasm, suggesting that circPDK1 may sponge miRNAs. To verify this hypothesis, miRNA-seq was performed and we found that miR-628-3p could be segregated by circPDK1. miR-628-3p has been indicated as an anti-oncogene in several cancers, such as lung cancer and glioblastoma, by modulating cell proliferation, apoptosis, migration, and invasion [44, 45]. However, the role of miR-628-3p in PC remains unknown. In this study, we found that miR-628-3p was downregulated in PC, and its low expression was associated with poor prognosis. It could also play an anti-tumor role in inhibiting proliferation, migration, and glycolysis by directly binding to BPTF in PC. BPTF, as a core subunit of the NURF chromatin-remodeling complex, is required for c-myc transcriptional activity, and the BPTF-c-myc axis is involved in cell growth in pancreatic cancer [33]. BPTF has been demonstrated to be an oncogene in various cancers, including renal cell carcinoma and glioma, through regulated glycolysis, metastasis, and proliferation [46, 47]. However, little research has been conducted on BPTF-mediated glycolysis and metastasis in PC. In our study, the effects of BPTF on migration and glycolysis were first confirmed in PC. The promotion of migration, proliferation, and glycolysis caused by miR-628-3p mimics could be eliminated by BPTF.

We demonstrated that circPDK1 degrades the stability of BIN1 by forming a circPDK1/BIN1/UBE2O triadic complex. In the literature, BIN1 has been found to inhibit tumor progression in various cancers by suppressing c-myc transcriptional activity [36, 48]. Nevertheless, little research has been conducted on ubiquitination degradation of the BIN1 protein. In the present study, we first established that circPDK1 could serve as a scaffold to recruit UBE2O, thus facilitating the UBE2O-induced ubiquitination degradation of BIN1 protein. circPDK1 was found to be essential for the highly efficient ubiquitination of BIN1 by UBE2O. In addition, UBE2O has been reported to accelerate tumor progression by modulating c-myc via the UBE2O/Mxi1 axis and UBE2O/AMPKα2/mTORC1 axis [49, 50]. Herein, we discovered another novel mechanism by which UBE2O modulates c-myc by the circPDK1/UBE2O/BIN1 ternary complex. Thus, our findings provide a novel mechanism for BIN1 protein degradation and novel insights into the pivotal role of circRNA in protein metabolism.

## Conclusions

We found that circPDK1 was highly abundant in exosomes derived from hypoxic PC cells. circPDK1 was upregulated in PC patients and serum exosomes, and high levels of circPDK1 expression were associated with a poor prognosis. Moreover, exosomal circPDK1 was shown to function as a tumor-promoting role in PC by enhancing growth, metastasis, and glycolysis both in vitro and in vivo. In addition, HIF1A upregulated circPDK1 by activating the host gene PDK1, and circPDK1 activated c-myc by modulating the miR-628-3p/BPTF axis and degrading BIN1. Thus, our research shows a model mechanism in which the circPDK1-c-myc axis promotes PC progression (Fig. 8J). This study provides novel insights into the multiplicity of circRNA/miRNA/mRNA and circRNA-RBP interactions and highlights its potential use as a biomarker and therapeutic target for PC.

## Supplementary Information

**Additional file 1: Table S1**. miRNA mimics or inhibitor sequences used in this study.

**Additional file 2: Table S2**. siRNA sequences used in this study.

**Additional file 3: Table S5** circPDK1 sequences used in this study

**Additional file 4: Table S3**. Antibodies used for Western blotting

**Additional file 5: Table S4**. Primer sequences used in this study

**Additional file 6.** Proteins pulled down by circPDK1

**Additional file 7: Figure S1**. Exosomes derived from hypoxia PC cells promote the tumorigenesis of PC cells in vitro*.* (A) Proposed schematic of intra-tumoral oxygen heterogeneity. (B) Representative transmission electron microscopy (TEM) images of exosomes in each group. Scale bar = 100 nm (C) Nanoparticle tracking analysis (NTA) was used to detect the exosome particle size and concentration. (D) exosome markers were detected by Western blotting. (E) MIA PaCa-2 and PANC-1 cells were cultured in 6-well plates after treated with indicated treatments. Scale bar = 1000 mm. (F) The EdU assay was performed to determine the cell proliferative potential of PC cells after treated with indicated exosomes. Scale bar = 50 μm. (G) The viabilities of PC cells were detected by CCK-8 assays after treated with indicated exosomes. (H) Transwell migration assay of MIA PaCa-2 and PANC-1 after treated with indicated exosomes. Scale bar = 50 μm. (I) The expression of metastasis-related proteins was evaluated by Western blotting after treated with indicated exosomes.

**Additional file 8: Table S6** Correlations between circPDK1 expression and clinical characteristics in PC patients

**Additional file 9: Table S7** Univariate and multivariate analysis of clinic pathological factors for overall survival in PC patients

**Additional file 10: Figure S2**. The expression of circPDK1 after treated with indicated treatments. (A) RNA abundance of circPDK1 after treatment with Actinomycin D. (B) RNA abundance of circPDK1 in normoxic exosomes, hypoxic exosomes, hypoxic NC exosomes and hypoxic sh1-circPDK1 exosomes. (C) RNA abundance of circPDK1 in MIA PaCa-2 and PANC-1 after treated with indicated exosomes. (D) RNA abundance of circPDK1 in MIA PaCa-2 and PANC-1 after transfected with circPDK1-overexpressing plasmids. *P < 0.05; ***P < 0.001; ns, no significance.

**Additional file 11: Figure S3**. The correction between potential target genes and miR-628-3p. (A) The correction between ARSJ and miR-628-3p were determined from TCGA. (B) The correction between PURA and miR-628-3p were determined from TCGA. (C) The correction between RBFOX2 and miR-628-3p were determined from TCGA.

**Additional file 12: Figure S4**. Cluster heatmap of differentially expressed protein-coding genes between control PANC-1 cells and those with lost circPDK1.

**Additional file 13: Figure S5**. miR-628-3p-BPTF axis participates in the tumor-promoting effects of circPDK1 in PC cells. (A) CCK-8 assay and (B) colony formation assay were used to detect the viabilities of MIA PaCa-2 cells transfected with circPDK1, miR-628-3p mimics or co-transfected with circPDK1 and miR-628-3p mimics (another group was transfected with miR-628-3p mimics or co-transfected with miR-628-3p mimics and BPTF). (C) Transwell migration assay was evaluated in MIA PaCa-2 cells with the same treatments. (D) ECAR, OCAR, (E) lactate production, and (F) glucose uptake assays were performed to measured glycolysis level in MIA PaCa-2 cells with indicated treatments. *P < 0.05; **P < 0.01; ***P < 0.001.

**Additional file 14: Figure S6**. Cytoplasmic co-localization of circPDK1, BIN1, and UBE2O. (A) Fluorescence intensity of circPDK1 and BIN1 protein. (B) Fluorescence intensity of circPDK1 and BIN1 and UBE2O proteins.

**Additional file 15: Figure S7**. BIN1 RNA expression was not affected by and circPDK1. BIN1 and circPDK1 RNA expression were not affected by UBE2O. (A) Expression of BIN1 RNA after loss of circPDK1 or circPDK1-overexpression. (B, C) Expression of BIN1 and circPDK1 RNA after loss of UBE2O. ns, not significant.

**Additional file 16: Figure S8**. Relative RNA expression of truncations after treatment with RNase R in 293 T cells, which were transfected with indicated truncation plasmids. **P < 0.01; ***P < 0.001.

**Additional file 17: Figure S9**. The mutations and back-splice junction site of circPDK1-MUT were verified using Sanger sequencing.

**Additional file 18: Figure S10**. BIN1 is a functional downstream mediator of circPDK1. (A) CCK-8 assay and (B) colony formation assays were used to detect the viabilities of MIA PaCa-2 cells transfected with circPDK1-WT, circPDK1-MUT, BIN1 or co-transfected with circPDK1-WT and BIN1. Scale bar = 1000 mm. (C) Transwell migration assays were used to measure the migration abilities after treated with the same treatments. Scale bar = 50 μm. (D) ECAR, OCAR, (E) lactate production, and (F) glucose uptake assays were performed to evaluate glycolysis level in MIA PaCa-2 cells with indicated treatments.

**Additional file 19: Figure S11**. circPDK1 promotes PC cells proliferation and metastasis in vivo. (A) Images of subcutaneous tumors. (B) The volume of subcutaneous tumors was calculated every six days. (C) The weight of subcutaneous tumors in each group. (D) Representative photographs of the whole lung tissues and HE staining of lung metastatic nodules. (E) Representative photographs of PCNA, E-cadherin, vimentin, PDK1, LDHA and c-myc IHC staining in subcutaneous tumors. Scale bar = 50 μm. *P < 0.05; **P < 0.01; ***P < 0.001; ns, no significance.

**Additional file 20: Figure S12**. UBE2O promoted PC tumorigenesis through degraded BIN1 protein and would be recruited by circPDK1. (A) Silver staining of circPDK1-associated proteins. (B) CCK-8 assay and (C) colony formation assays were used to measure the proliferation of MIA PaCa-2 cells transfected with UBE2O or co-transfected with UBE2O and BIN1, or knocked down circPDK1 while UBE2O overexpression. Scale bar = 1000 mm. (D) Transwell migration assays were performed to evaluate the migration abilities after treated with the same treatments. Scale bar = 50 μm. (E) ECAR, OCAR, (F) lactate production and (G) glucose uptake were performed to evaluate glycolysis level in MIA PaCa-2 cells with indicated treatments. *P < 0.05; **P < 0.01; ***P < 0.001.

**Additional file 21: Figure S13**. circPDK1 activates c-myc through two non-interfering axes. miR-628-3p or BPTF expressions were detected after BIN1 overexpression or loss of UBE2O by (A-B) qRT-PCR and (C) Western blotting. UBE2O or BIN1 expressions were measured after treatments with miR-628-3p mimics or inhibitor, and BPTF overexpression in (D) qRT-PCR and (E) Western blotting. ns, no significance.

## Data Availability

The datasets supporting the conclusions of this article are included within the article and its additional files.

## Abbreviations

PC: Pancreatic cancer
circRNA: Circular RNA
ceRNA: Competitive endogenous RNA
qRT-PCR: Quantitative reverse transcription polymerase chain reaction
RIP: RNA immunoprecipitation
FISH: Fluorescent in situ hybridization
IF: Immunofluorescence
CCK-8: Cell count kit-8
EdU: 5-Ethynyl-20-deoxyuridine
AGO2: Argonaute-2
IHS: In situ hybridization
Co-IP: Co-immunoprecipitation
IHC: Immunohistochemistry
HE: Hematoxylin–eosin
ChIP: Chromatin immunoprecipitation
ECAR: Extracellular acidification rate
OCAR: Oxygen consumption rate
SD: Standard deviation
