# Supplementary material for: Hypoxia-induced exosomal circPDK1 promotes pancreatic cancer glycolysis via c-myc activation by modulating miR-628-3p/BPTF axis and degrading BIN1
Source: J Hematol Oncol. 2022 Sep 6;15:128. doi: 10.1186/s13045-022-01348-7 (PMC9450374; doi:10.1186/s13045-022-01348-7)
Supplement: Supplementary file 1 — Additional file 1: Table S1. miRNA mimics or inhibitor sequences used in this study. [file 13045_2022_1348_MOESM1_ESM.docx]

**Additional file 1: Table S1**. miRNA mimics or inhibitor sequences used in this study

| miR-628-3p sense | 5′-UCUAGUAAGAGUGGCAGUCGA-3′ |
| --- | --- |
| miR-628-3p anti-sense | 5′-UCGACUGCCACUCUUACUAGA-3′ |
| miR-NC sense | 5′-UUCUCCGAACGUGUCACGUTT-3′ |
| miR-NC anti-sense | 5′-ACGUGACACGUUCGGAGAATT-3′ |
| miR-628-3p inhibitor | 5′-UCGACUGCCACUCUUACUAGA-3′ |
