# Supplementary material for: Hypoxia-induced exosomal circPDK1 promotes pancreatic cancer glycolysis via c-myc activation by modulating miR-628-3p/BPTF axis and degrading BIN1
Source: J Hematol Oncol. 2022 Sep 6;15:128. doi: 10.1186/s13045-022-01348-7 (PMC9450374; doi:10.1186/s13045-022-01348-7)
Supplement: Supplementary file 2 — Additional file 2: Table S2. siRNA sequences used in this study. [file 13045_2022_1348_MOESM2_ESM.docx]

**Additional file 2: Table S2.** siRNA sequences used in this study

| si-circPDK1#1 | CUACAUUAAGCAAAAUCACCATT |
| --- | --- |
| si-circPDK1#2 | UACAUUAAGCAAAAUCACCAGTT |
| siRNA-NC | UUCUCCGAACGUGUCACGUTT |
| si-UBE2O#1 | GCACCAACTGCATCATCTATC |
| si-UBE2O#2 | GGTTGTAGAGTTGAAAGTTAC |
| si-HIF1A | GGGATTAACTCAGTTTGAACT |
