# Supplementary material for: Hypoxia-induced exosomal circPDK1 promotes pancreatic cancer glycolysis via c-myc activation by modulating miR-628-3p/BPTF axis and degrading BIN1
Source: J Hematol Oncol. 2022 Sep 6;15:128. doi: 10.1186/s13045-022-01348-7 (PMC9450374; doi:10.1186/s13045-022-01348-7)
Supplement: Supplementary file 3 — Additional file 3: Table S5 circPDK1 sequences used in this study [file 13045_2022_1348_MOESM3_ESM.docx]

**Additional file 3: Table S5** circPDK1 sequences used in this study

| circPDK1-WT sequences |
| --- |
| CAAAATCACCAGGACAGCCAATACAAGTGGTTTATGTACCATCCCATCTCTATCACATGGTGTTTGAACTTTTCAAGAATGCAATGAGAGCCACTATGGAACACCATGCCAACAGAGGTGTTTACCCCCCTATTCAAGTTCATGTCACGCTGGGTAATGAGGATTTGACTGTGAAGATGAGTGACCGAGGAGGTGGCGTTCCTTTGAGGAAAATTGACAGACTTTTCAACTACATGTATTCAACTGCACCAAGACCTCGTGTTGAGACCTCCCGCGCAGTGCCTCTGGCTGGTTTTGGTTATGGATTGCCCATATCACGTCTTTACGCACAATACTTCCAAGGAGACCTGAAGCTGTATTCCCTAGAGGGTTACGGGACAGATGCAGTTATCTACATTAAG |
| circPDK1-MUT sequences |
| CAAAATCACCAGGACAGCCAATACAAGTGGTTTATGTACCATCCCATCTCTATCACATGGTGTTTGAACTTTTCAAGAATGCAATGAGAGCCACTATGGAACACCATGCCAACAGAGGTGTTTACCCCCCTATTCAAGTTCATGTCACGCTGGGTAATGAGGATTTGACTGTGAAGATGAGTGACCGAGGAGGTGGCGTTCCTTTGAGGAAAATTGACAGACTTTTCAACTACATGTATTCAACACGTGGAAGACCTCGTGTTGAGACCTCCCGCGCAGTGCCTCTGGCTGGTTTTGGTTATGGATTGCGGTATACACGTCTTTACGCACAATACTTCCAAGGAGACCTGAAGCTGTATTCCCTAGAGGGTTACGGGACAGATGCAGTTATCTACATTAAG |
