# Supplementary material for: Hypoxia-induced exosomal circPDK1 promotes pancreatic cancer glycolysis via c-myc activation by modulating miR-628-3p/BPTF axis and degrading BIN1
Source: J Hematol Oncol. 2022 Sep 6;15:128. doi: 10.1186/s13045-022-01348-7 (PMC9450374; doi:10.1186/s13045-022-01348-7)
Supplement: Supplementary file 4 — Additional file 4: Table S3. Antibodies used for Western blotting [file 13045_2022_1348_MOESM4_ESM.docx]

| **Additional file 4: Table S3**. Antibodies used for western blotting | | | | |
| --- | --- | --- | --- | --- |
| **Antibody** | **Company** | **Cat. No.** | **Species** | **Dilution** |
| BPTF | Abcam | ab288159 | Rabbit | 1:1000 |
| β-actin | Cell signaling | 4970 | Rabbit | 1:1000 |
| β-tubulin | Proteintech | 10068-1-AP | Rabbit | 1:1000 |
| E-cadherin | Cell signaling | 3195 | Rabbit | 1:1000 |
| N-cadherin | Cell signaling | 13116 | Rabbit | 1:1000 |
| vimentin | Cell signaling | 5741 | Rabbit | 1:1000 |
| c-myc | Cell signaling | 18583 | Rabbit | 1:1000 |
| HIF1A | Cell signaling | 36169 | Rabbit | 1:1000 |
| BIN1 | Abcam | ab182562 | Rabbit | 1:1000 |
| FLAG-Tag | Proteintech | 20543-1-AP | Rabbit | 1:2000 |
| UBE2O | Abcolnal | A10036 | Rabbit | 1:1000 |
| MYC-Tag | Proteintech | 16286-1-AP | Rabbit | 1:2000 |
| Ubiquitin | Cell signaling | 3933 | Rabbit | 1:2000 |
| HK2 | Abcolnal | A0994 | Rabbit | 1:1000 |
| GLUT1 | Abcolnal | A11727 | Rabbit | 1:1000 |
| PDK1 | Proteintech | 18262-1-AP | Rabbit | 1:1000 |
| PKM2 | Proteintech | 25659-1-AP | Rabbit | 1:1000 |
| LDHA | Abcolnal | A0861 | Rabbit | 1:1000 |
| CCND1 | Abcolnal | A19038 | Rabbit | 1:1000 |
| P21 | Abcolnal | A21061 | Rabbit | 1:1000 |
