# Supplementary material for: Hypoxia-induced exosomal circPDK1 promotes pancreatic cancer glycolysis via c-myc activation by modulating miR-628-3p/BPTF axis and degrading BIN1
Source: J Hematol Oncol. 2022 Sep 6;15:128. doi: 10.1186/s13045-022-01348-7 (PMC9450374; doi:10.1186/s13045-022-01348-7)
Supplement: Supplementary file 5 — Additional file 5: Table S4. Primer sequences used in this study [file 13045_2022_1348_MOESM5_ESM.docx]

**Additional file 5: Table S4**. Primer sequences used in this study

| Primers used for qPCR: | |
| --- | --- |
| circPDK1 forward | CATTAAGCAAAATCACCAGGACA |
| circPDK1 reverse | TACCCAGCGTGACATGAACT |
| β-actin forward | CATGTACGTTGCTATCCAGGC |
| β-actin reverse | CTCCTTAATGTCACGCACGAT |
| miR-628-3p forward | GGCGGCTCTAGTAAGAGTGGC |
| miR-628-3p reverse | ACTGCAGGGTCCGAGGTATT |
| U6 forward | CTCGCTTCGGCAGCACA |
| U6 reverse | AACGCTTCACGAATTTGCGT |
| BIN1 forward | GCAGGGATGAGGCAAACAAG |
| BIN1 reverse | CACTGTCGTAGTCCACCAGC |
| UBE2O forward | TCCAACGACCTCTTCCCTGT |
| UBE2O reverse | GTACCACACCGTAGACAGCA |
| HIF1A forward | TGAACCCATTCCTCACCCATC |
| HIF1A reverse | CCTGTACTGTCCTGTGGTGA |
| BPTF forward | CTGCTGCTTCCCAGAAGAGG |
| BPTF reverse | GCCTCACTTTGCAAGATGCC |
| GAPDH divergent forward | CCCTGTGCTCAACCAGCTCTC |
| GAPDH divergent reverse | CCGACCTTCACCTTCCCCAT |
| GAPDH convergent forward | GGAGTCCACTGGCGTCTTCA |
| GAPDH convergent reverse | GTCATGAGTCCTTCCACGATACC |
| circPDK1 convergent forward | GCCACTATGGAACACCATGC |
| circPDK1 convergent reverse | ACCTCCTCGGTCACTCATCT |
| Truncation1 1-100bp forward | TCACCAGGACAGCCAATACA |
| Truncation1 1-100bp reverse | TCCATAGTGGCTCTCATTGCAT |
| Truncation2 101-200bp forward | AAGTTCATGTCACGCTGGGT |
| Truncation2 101-200bp reverse | CACCTCCTCGGTCACTCATC |
| Truncation3 201-300bp forward | TCAACTGCACCAAGACCTCG |
| Truncation3 201-300bp reverse | AACCAAAACCAGCCAGAGGC |
| Truncation4 301-401bp forward | GCCCATATCACGTCTTTACGC |
| Truncation4 301-401bp reverse | GCATCTGTCCCGTAACCCTC |
| Primers used for ChIP |  |
| HRE1 forward | TATAGATTGAGGTCTCCTGTCTCC |
| HRE1 reverse | GTGAACTCCGAAAGTTTGAGAC |
| HRE2 forward | CGCCCGCCCCTTTTCTCTC |
| HRE2 reverse | CTAGAGAAGCCACAGCCAGTACG |
| PDK1 promoter forward | CACACGTCCCAGTGTTCAGCTTTC |
| PDK1 promoter reverse | AGAAACGCTCCTCTGTGACGCAAG |
