# Supplementary figures and images for: Hypoxia-induced exosomal circPDK1 promotes pancreatic cancer glycolysis via c-myc activation by modulating miR-628-3p/BPTF axis and degrading BIN1
Source: J Hematol Oncol. 2022 Sep 6;15:128. doi: 10.1186/s13045-022-01348-7 (PMC9450374; doi:10.1186/s13045-022-01348-7)

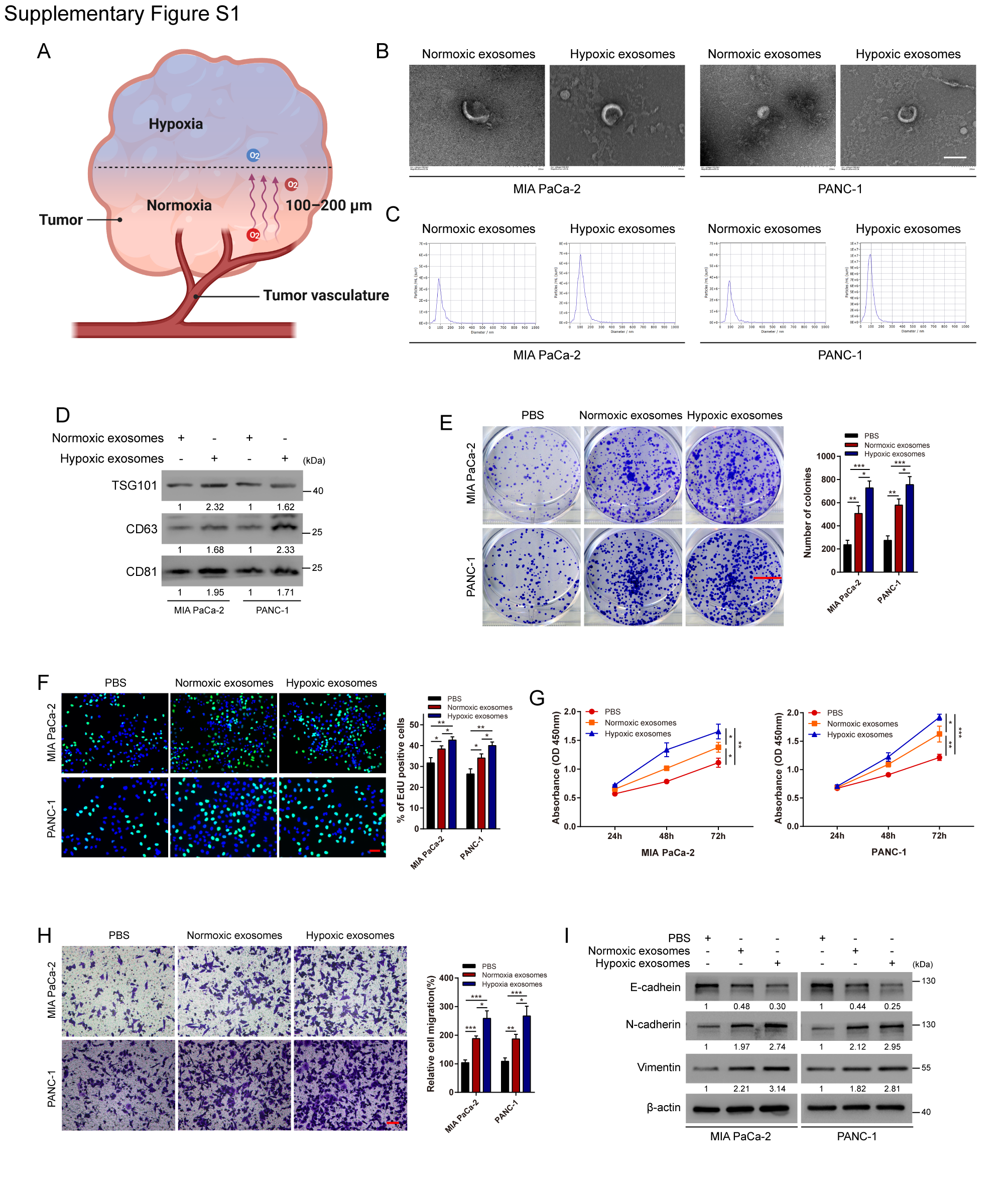

Supplement: Supplementary file 7 — Additional file 7: Figure S1. Exosomes derived from hypoxia PC cells promote the tumorigenesis of PC cells in vitro. (A) Proposed schematic of intra-tumoral oxygen heterogeneity. (B) Representative transmission electron microscopy (TEM) images of exosomes in each group. Scale bar = 100 nm (C) Nanoparticle tracking analysis (NTA) was used to detect the exosome particle size and concentration. (D) exosome markers were detected by Western blotting. (E) MIA PaCa-2 and PANC-1 cells were cultured in 6-well plates after treated with indicated treatments. Scale bar = 1000 mm. (F) The EdU assay was performed to determine the cell proliferative potential of PC cells after treated with indicated exosomes. Scale bar = 50 μm. (G) The viabilities of PC cells were detected by CCK-8 assays after treated with indicated exosomes. (H) Transwell migration assay of MIA PaCa-2 and PANC-1 after treated with indicated exosomes. Scale bar = 50 μm. (I) The expression of metastasis-related proteins was evaluated by Western blotting after treated with indicated exosomes. [file 13045_2022_1348_MOESM7_ESM.tif]

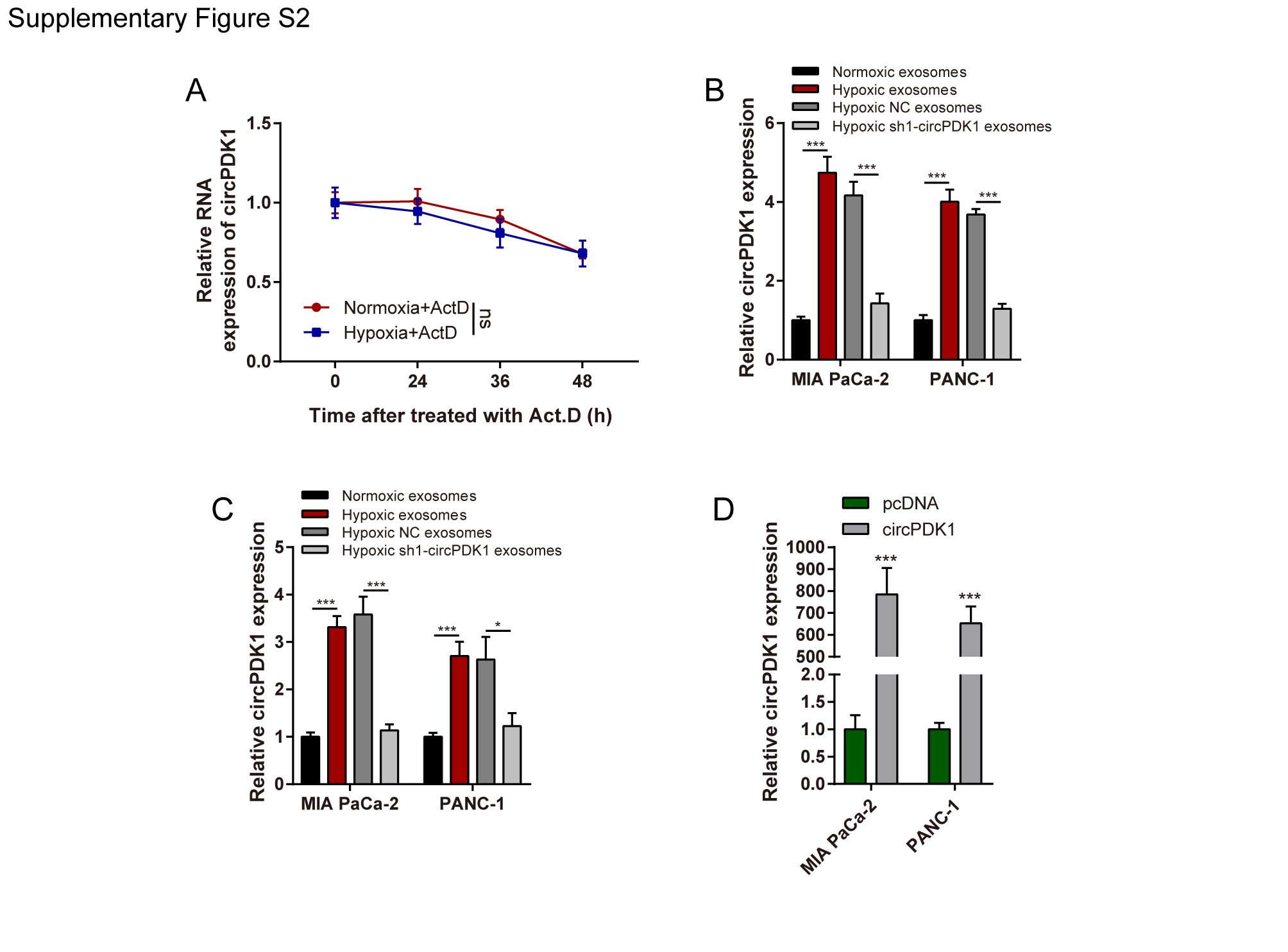

Supplement: Supplementary file 10 — Additional file 10: Figure S2. The expression of circPDK1 after treated with indicated treatments. (A) RNA abundance of circPDK1 after treatment with Actinomycin D. (B) RNA abundance of circPDK1 in normoxic exosomes, hypoxic exosomes, hypoxic NC exosomes and hypoxic sh1-circPDK1 exosomes. (C) RNA abundance of circPDK1 in MIA PaCa-2 and PANC-1 after treated with indicated exosomes. (D) RNA abundance of circPDK1 in MIA PaCa-2 and PANC-1 after transfected with circPDK1-overexpressing plasmids. *P < 0.05; ***P < 0.001; ns, no significance. [file 13045_2022_1348_MOESM10_ESM.tif]

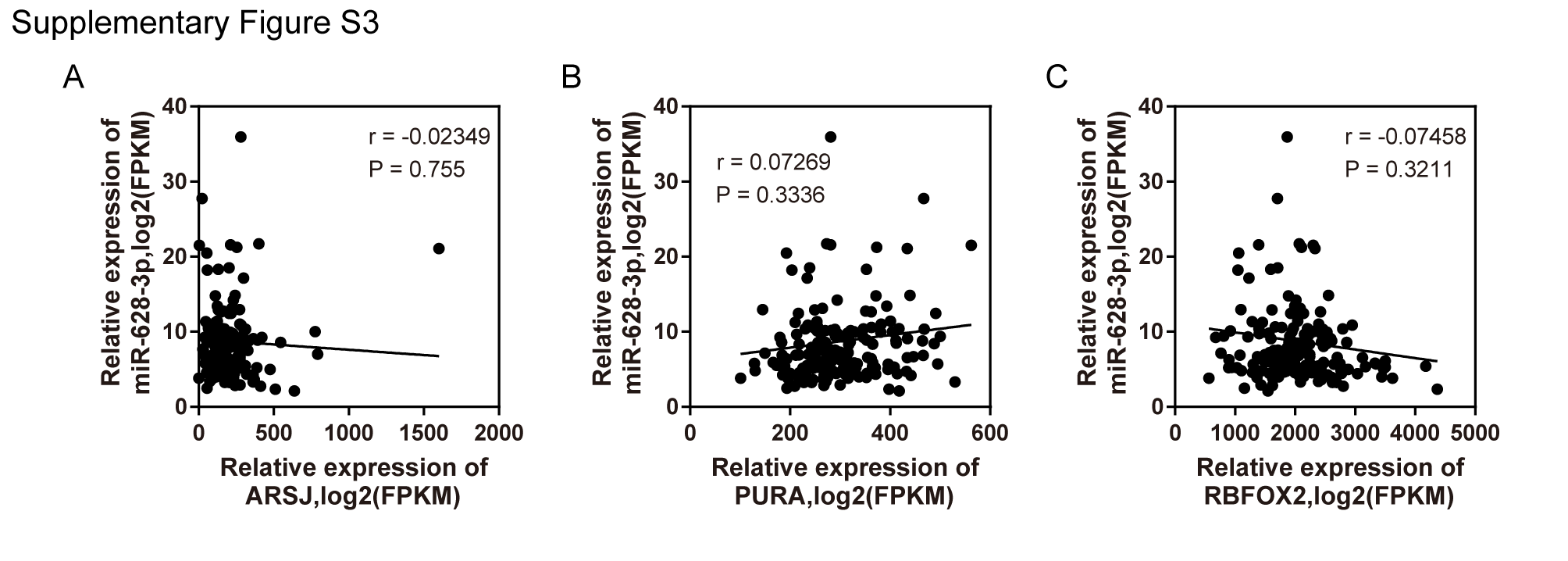

Supplement: Supplementary file 11 — Additional file 11: Figure S3. The correction between potential target genes and miR-628-3p. (A) The correction between ARSJ and miR-628-3p were determined from TCGA. (B) The correction between PURA and miR-628-3p were determined from TCGA. (C) The correction between RBFOX2 and miR-628-3p were determined from TCGA. [file 13045_2022_1348_MOESM11_ESM.tif]

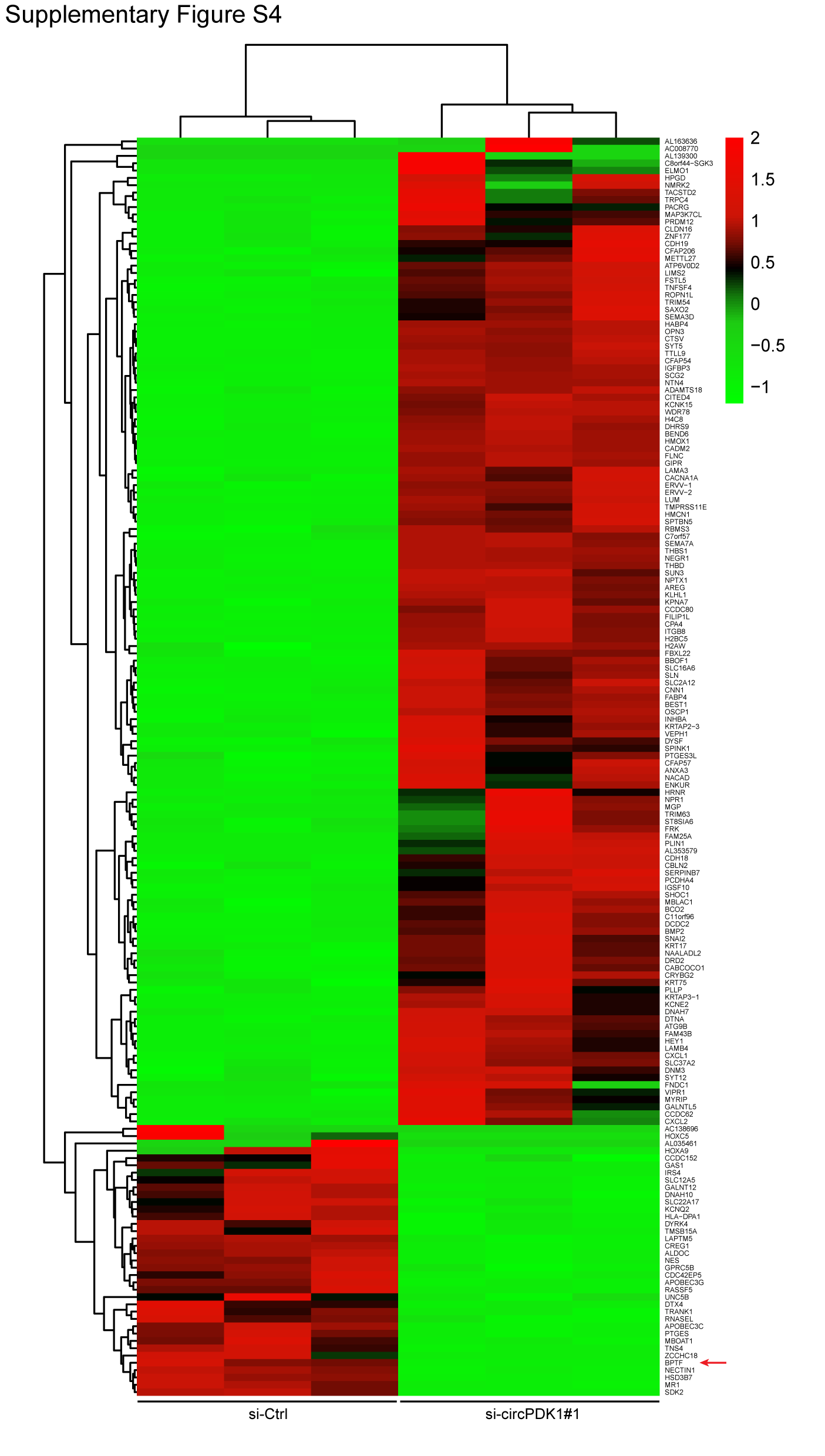

Supplement: Supplementary file 12 — Additional file 12: Figure S4. Cluster heatmap of differentially expressed protein-coding genes between control PANC-1 cells and those with lost circPDK1. [file 13045_2022_1348_MOESM12_ESM.tif]

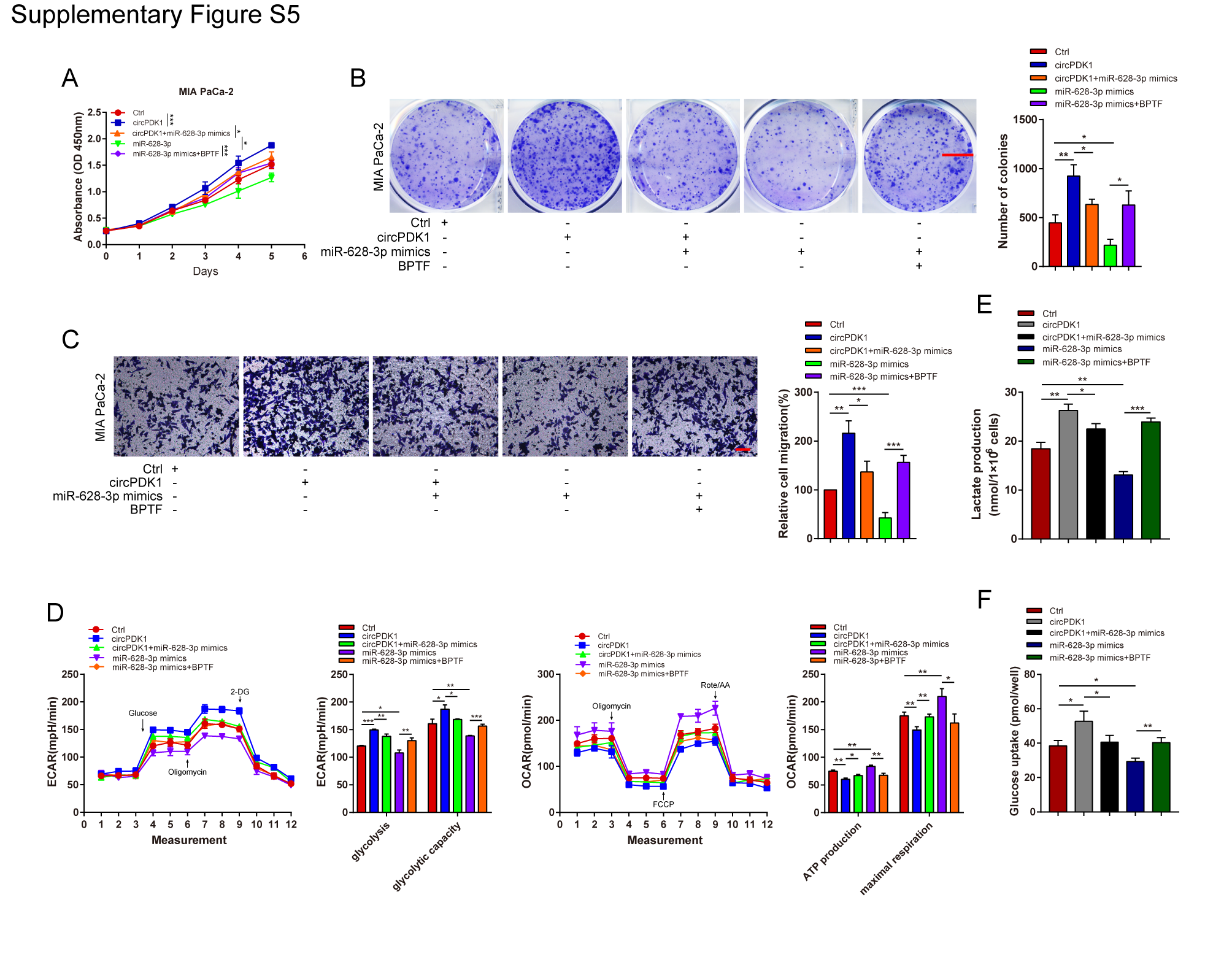

Supplement: Supplementary file 13 — Additional file 13: Figure S5. miR-628-3p-BPTF axis participates in the tumor-promoting effects of circPDK1 in PC cells. (A) CCK-8 assay and (B) colony formation assay were used to detect the viabilities of MIA PaCa-2 cells transfected with circPDK1, miR-628-3p mimics or co-transfected with circPDK1 and miR-628-3p mimics (another group was transfected with miR-628-3p mimics or co-transfected with miR-628-3p mimics and BPTF). (C) Transwell migration assay was evaluated in MIA PaCa-2 cells with the same treatments. (D) ECAR, OCAR, (E) lactate production, and (F) glucose uptake assays were performed to measured glycolysis level in MIA PaCa-2 cells with indicated treatments. *P < 0.05; **P < 0.01; ***P < 0.001. [file 13045_2022_1348_MOESM13_ESM.tif]

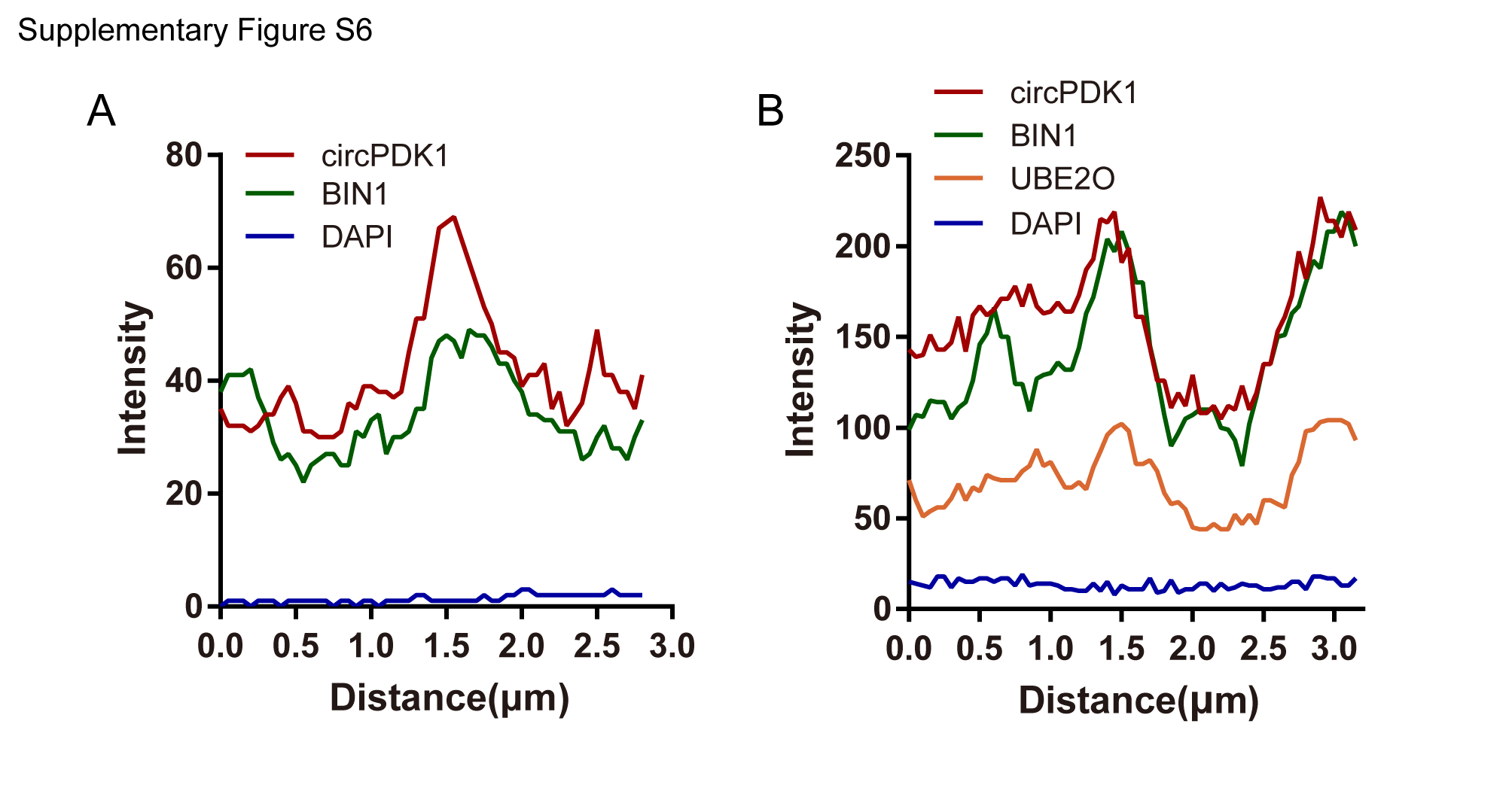

Supplement: Supplementary file 14 — Additional file 14: Figure S6. Cytoplasmic co-localization of circPDK1, BIN1, and UBE2O. (A) Fluorescence intensity of circPDK1 and BIN1 protein. (B) Fluorescence intensity of circPDK1 and BIN1 and UBE2O proteins. [file 13045_2022_1348_MOESM14_ESM.tif]

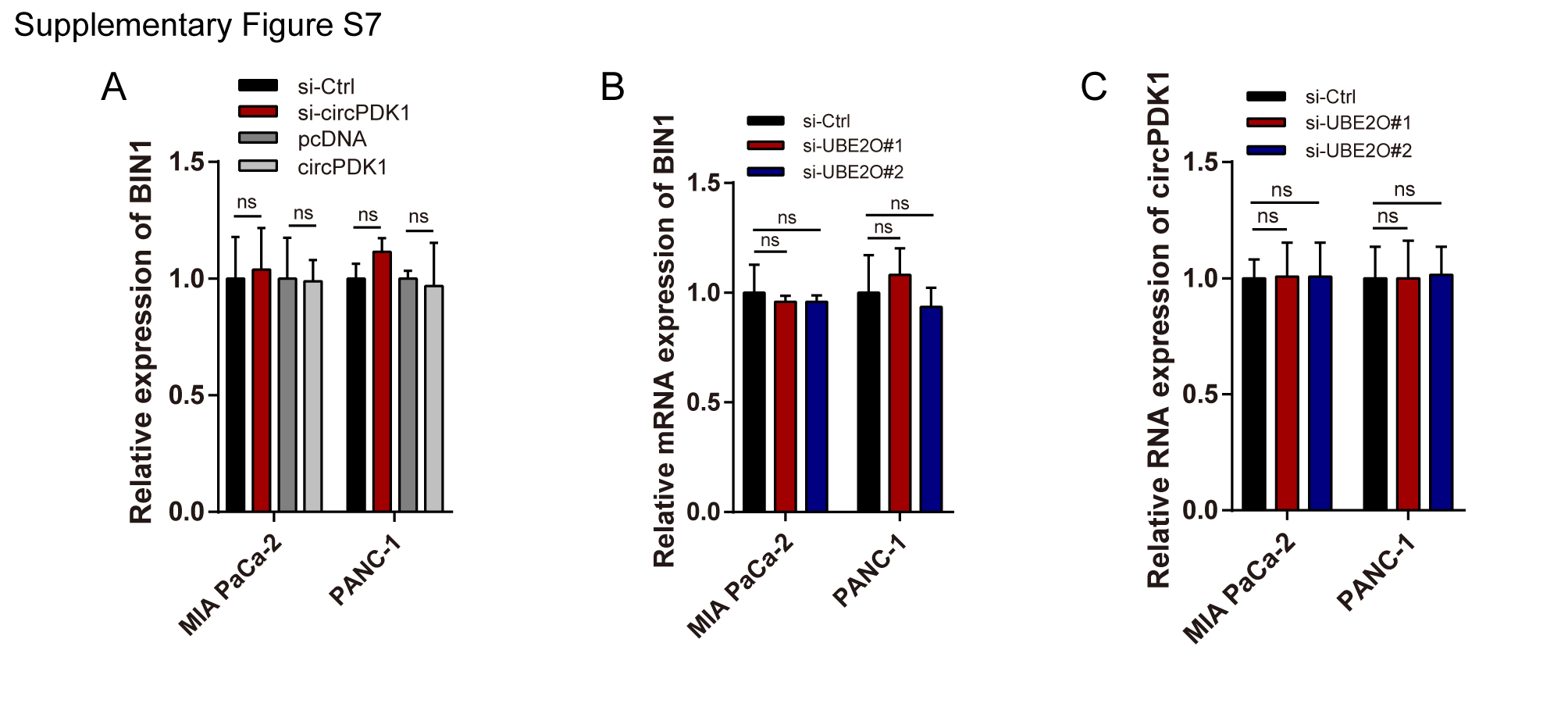

Supplement: Supplementary file 15 — Additional file 15: Figure S7. BIN1 RNA expression was not affected by and circPDK1. BIN1 and circPDK1 RNA expression were not affected by UBE2O. (A) Expression of BIN1 RNA after loss of circPDK1 or circPDK1-overexpression. (B, C) Expression of BIN1 and circPDK1 RNA after loss of UBE2O. ns, not significant. [file 13045_2022_1348_MOESM15_ESM.tif]

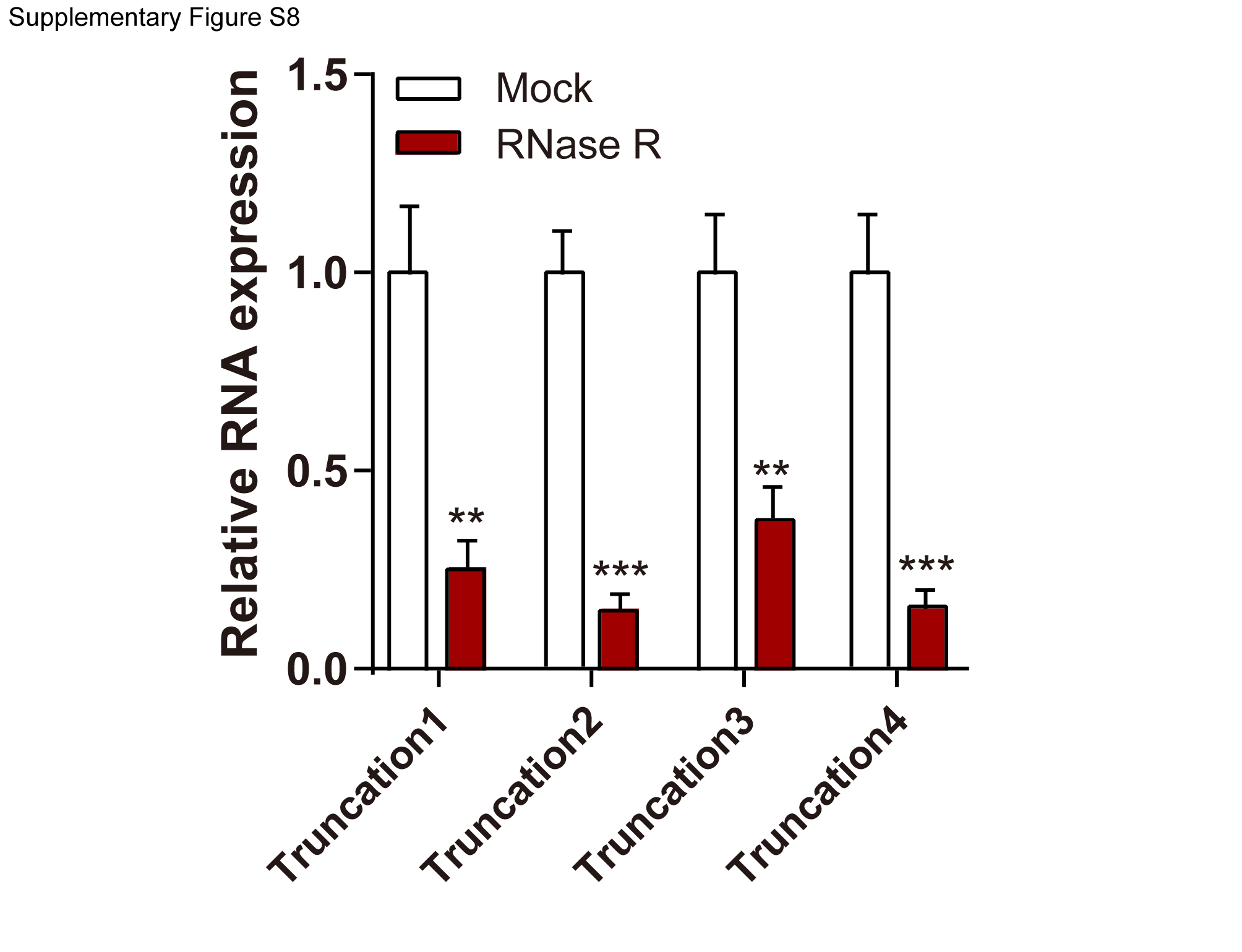

Supplement: Supplementary file 16 — Additional file 16: Figure S8. Relative RNA expression of truncations after treatment with RNase R in 293 T cells, which were transfected with indicated truncation plasmids. **P < 0.01; ***P < 0.001. [file 13045_2022_1348_MOESM16_ESM.tif]

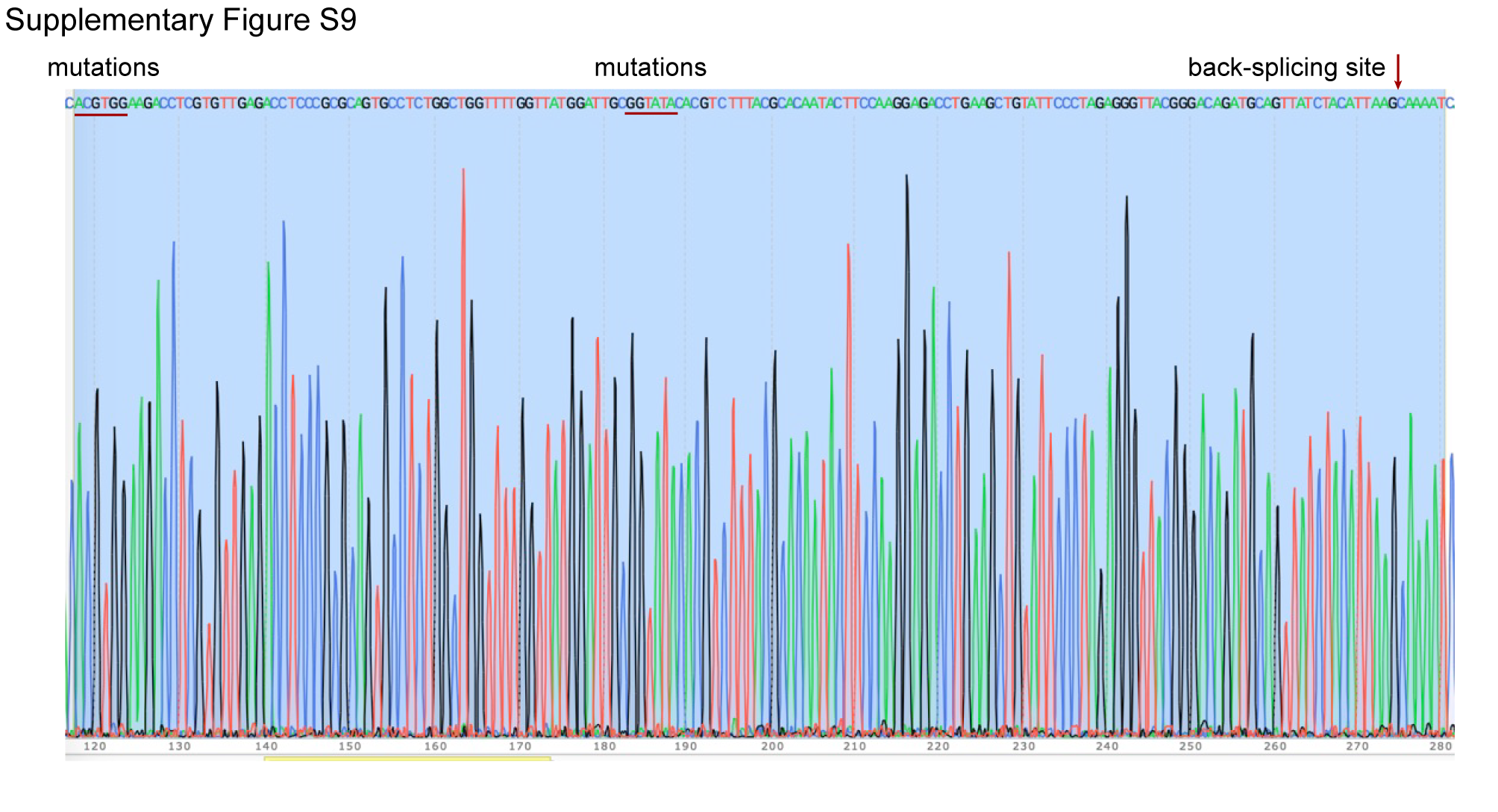

Supplement: Supplementary file 17 — Additional file 17: Figure S9. The mutations and back-splice junction site of circPDK1-MUT were verified using Sanger sequencing. [file 13045_2022_1348_MOESM17_ESM.tif]

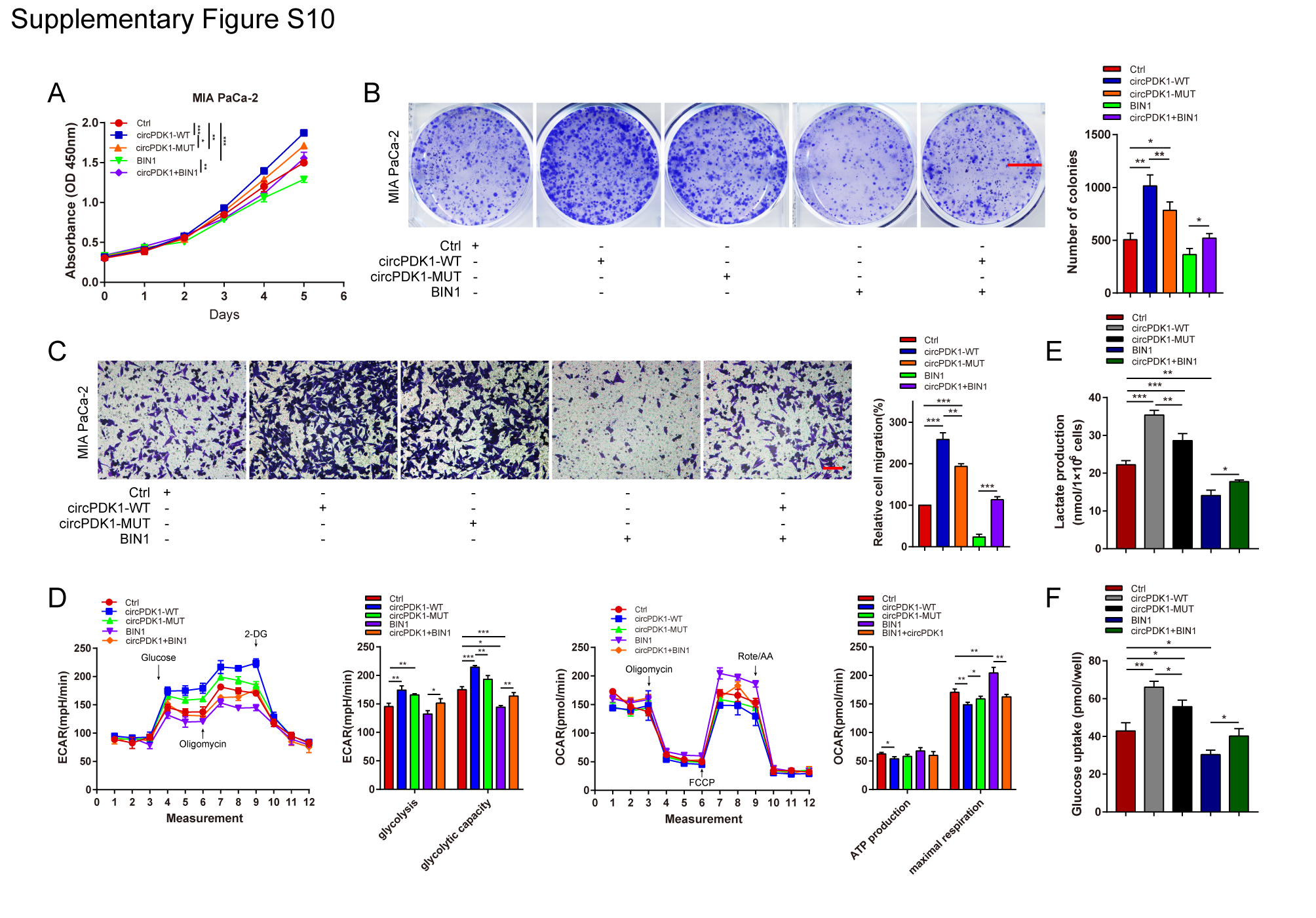

Supplement: Supplementary file 18 — Additional file 18: Figure S10. BIN1 is a functional downstream mediator of circPDK1. (A) CCK-8 assay and (B) colony formation assays were used to detect the viabilities of MIA PaCa-2 cells transfected with circPDK1-WT, circPDK1-MUT, BIN1 or co-transfected with circPDK1-WT and BIN1. Scale bar = 1000 mm. (C) Transwell migration assays were used to measure the migration abilities after treated with the same treatments. Scale bar = 50 μm. (D) ECAR, OCAR, (E) lactate production, and (F) glucose uptake assays were performed to evaluate glycolysis level in MIA PaCa-2 cells with indicated treatments. [file 13045_2022_1348_MOESM18_ESM.tif]

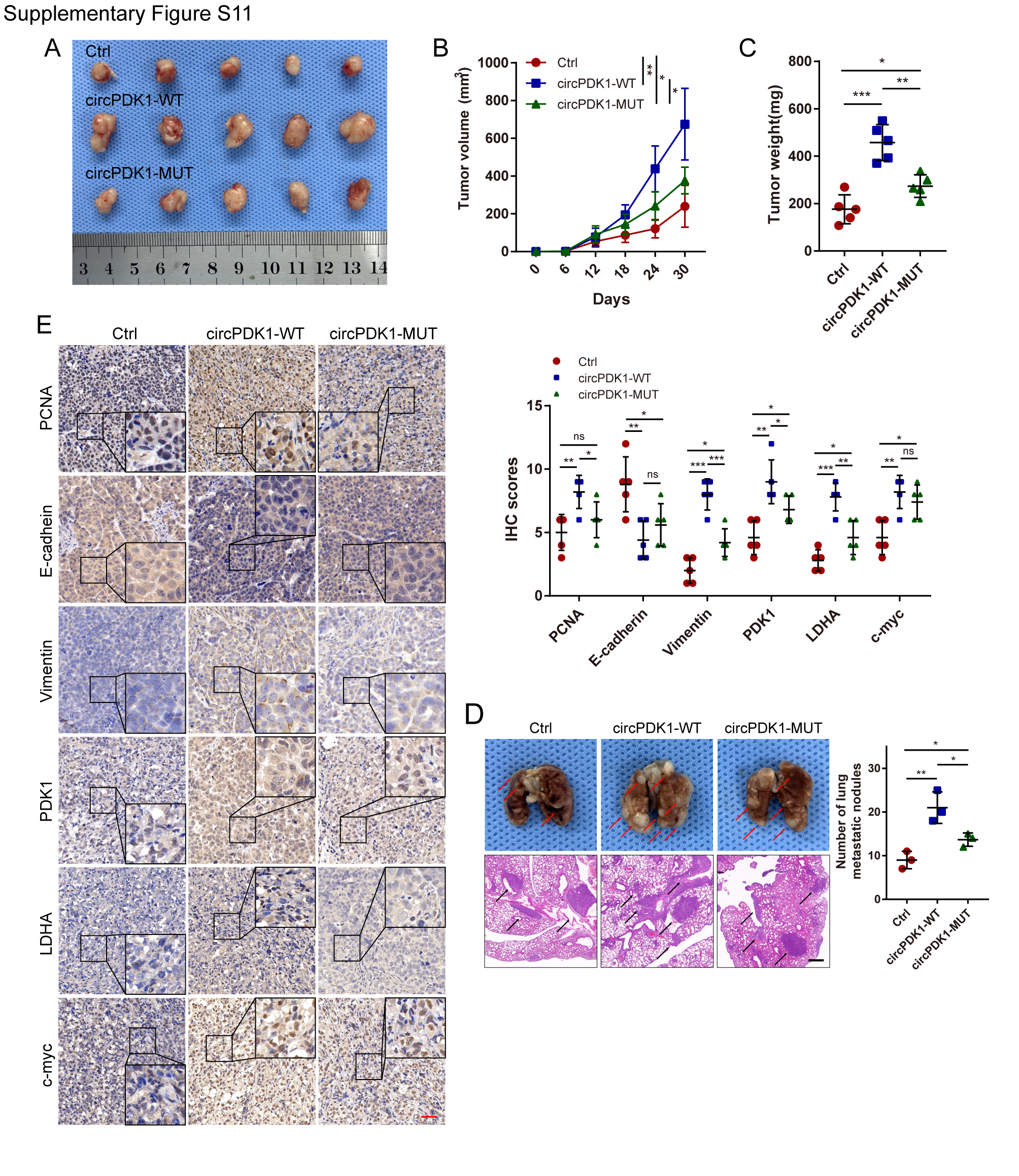

Supplement: Supplementary file 19 — Additional file 19: Figure S11. circPDK1 promotes PC cells proliferation and metastasis in vivo. (A) Images of subcutaneous tumors. (B) The volume of subcutaneous tumors was calculated every six days. (C) The weight of subcutaneous tumors in each group. (D) Representative photographs of the whole lung tissues and HE staining of lung metastatic nodules. (E) Representative photographs of PCNA, E-cadherin, vimentin, PDK1, LDHA and c-myc IHC staining in subcutaneous tumors. Scale bar = 50 μm. *P < 0.05; **P < 0.01; ***P < 0.001; ns, no significance. [file 13045_2022_1348_MOESM19_ESM.tif]

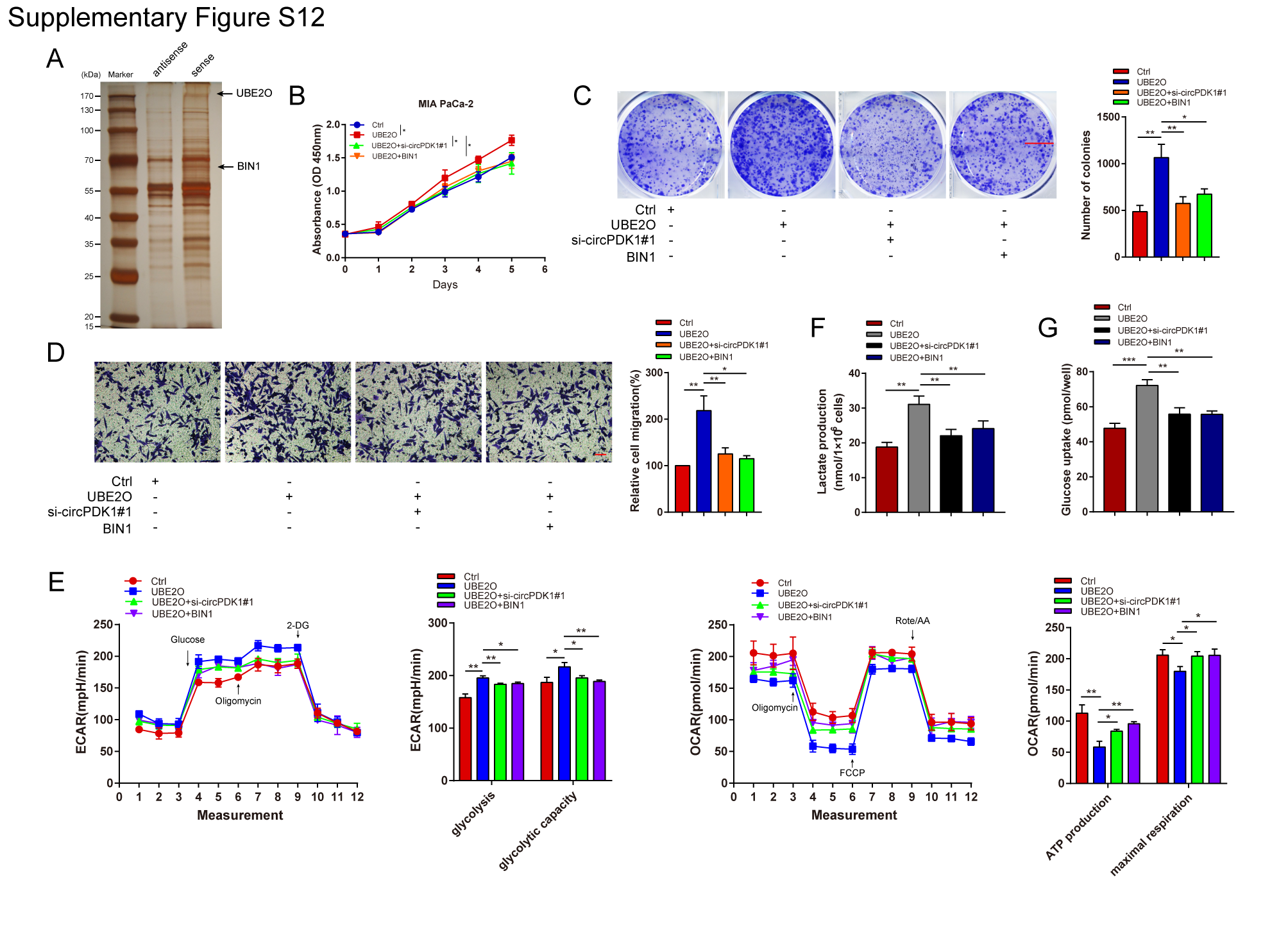

Supplement: Supplementary file 20 — Additional file 20: Figure S12. UBE2O promoted PC tumorigenesis through degraded BIN1 protein and would be recruited by circPDK1. (A) Silver staining of circPDK1-associated proteins. (B) CCK-8 assay and (C) colony formation assays were used to measure the proliferation of MIA PaCa-2 cells transfected with UBE2O or co-transfected with UBE2O and BIN1, or knocked down circPDK1 while UBE2O overexpression. Scale bar = 1000 mm. (D) Transwell migration assays were performed to evaluate the migration abilities after treated with the same treatments. Scale bar = 50 μm. (E) ECAR, OCAR, (F) lactate production and (G) glucose uptake were performed to evaluate glycolysis level in MIA PaCa-2 cells with indicated treatments. *P < 0.05; **P < 0.01; ***P < 0.001. [file 13045_2022_1348_MOESM20_ESM.tif]

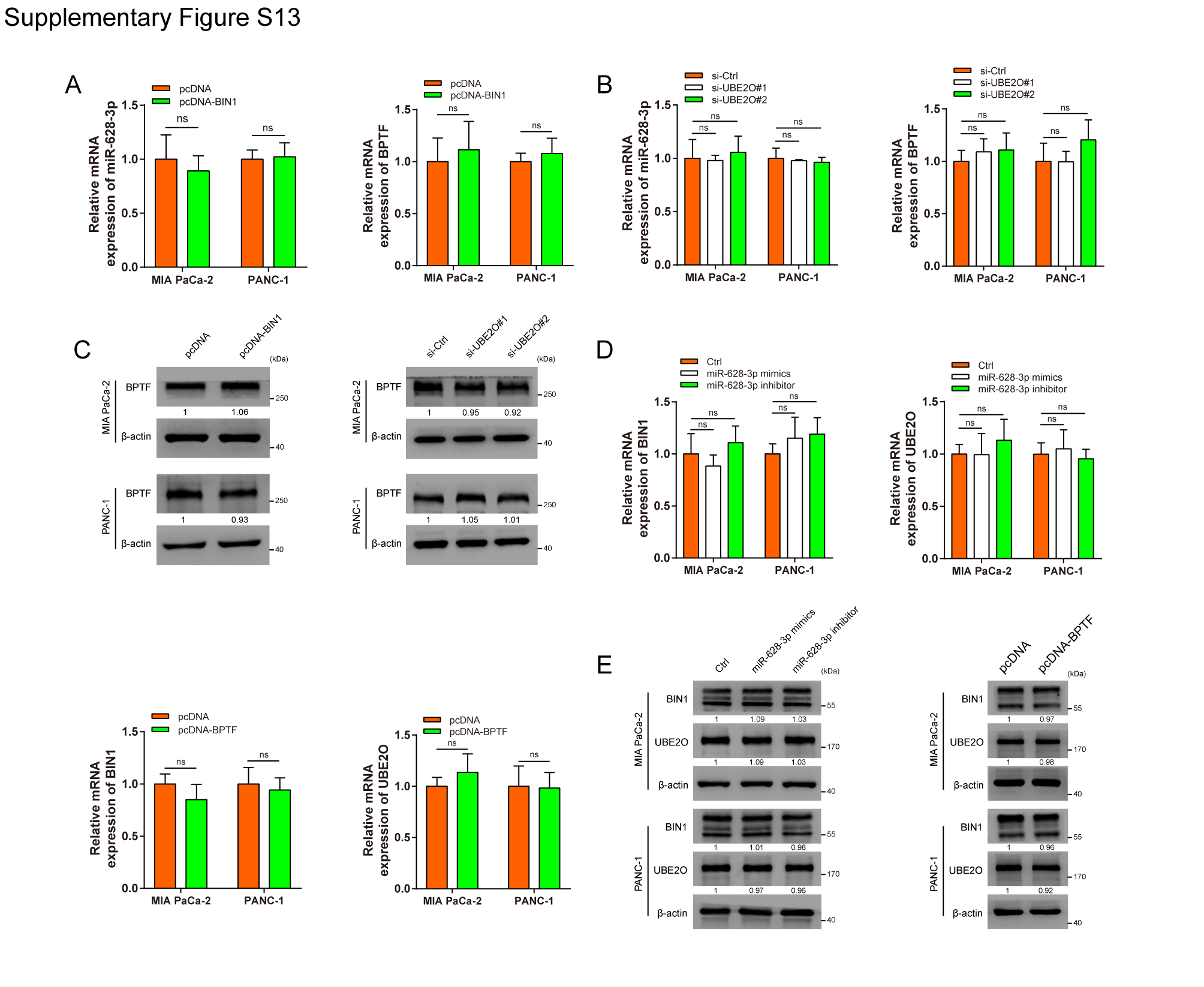

Supplement: Supplementary file 21 — Additional file 21: Figure S13. circPDK1 activates c-myc through two non-interfering axes. miR-628-3p or BPTF expressions were detected after BIN1 overexpression or loss of UBE2O by (A-B) qRT-PCR and (C) Western blotting. UBE2O or BIN1 expressions were measured after treatments with miR-628-3p mimics or inhibitor, and BPTF overexpression in (D) qRT-PCR and (E) Western blotting. ns, no significance. [file 13045_2022_1348_MOESM21_ESM.tif]
