# Supplementary material for: Hypoxia-induced exosomal circPDK1 promotes pancreatic cancer glycolysis via c-myc activation by modulating miR-628-3p/BPTF axis and degrading BIN1
Source: J Hematol Oncol. 2022 Sep 6;15:128. doi: 10.1186/s13045-022-01348-7 (PMC9450374; doi:10.1186/s13045-022-01348-7)
Supplement: Supplementary file 8 — Additional file 8: Table S6 Correlations between circPDK1 expression and clinical characteristics in PC patients [file 13045_2022_1348_MOESM8_ESM.docx]

| Clinicopathologic parameters | Case (n = 110) | circPDK1 expression | | *P value* |
| --- | --- | --- | --- | --- |
|  |  | Low | High |  |
| Total | 110 | 46 | 64 |  |
| Gender |  |  |  | 0.488 |
| Male | 70 | 31 | 39 |  |
| Female | 40 | 15 | 25 |  |
| Age |  |  |  | 0.391 |
| ≥ 60 | 65 | 25 | 40 |  |
| < 60 | 45 | 21 | 24 |  |
| Pathological stage |  |  |  | 0.019 |
| I-II | 75 | 37 | 38 |  |
| III-IV | 35 | 9 | 26 |  |
| T stage |  |  |  | 0.022 |
| T1-2 | 65 | 33 | 32 |  |
| T3-4 | 45 | 13 | 32 |  |
| Lymph node metastasis |  |  |  | 0.012 |
| N0 | 40 | 24 | 16 |  |
| N1 | 58 | 19 | 39 |  |
| N2 | 12 | 3 | 9 |  |
| Distant metastasis |  |  |  | 0.034 |
| M0 | 102 | 46 | 56 |  |
| M1 | 8 | 0 | 8 |  |

**Additional file 8: Table S6** Correlations between circPDK1 expression and clinical characteristics in PC patients
