# Supplementary material for: Hypoxia-induced exosomal circPDK1 promotes pancreatic cancer glycolysis via c-myc activation by modulating miR-628-3p/BPTF axis and degrading BIN1
Source: J Hematol Oncol. 2022 Sep 6;15:128. doi: 10.1186/s13045-022-01348-7 (PMC9450374; doi:10.1186/s13045-022-01348-7)
Supplement: Supplementary file 9 — Additional file 9: Table S7 Univariate and multivariate analysis of clinic pathological factors for overall survival in PC patients [file 13045_2022_1348_MOESM9_ESM.docx]

**Additional file 9: Table S7** Univariate and multivariate analysis of clinic pathological factors for overall survival in PC patients

| Variables | Univariate Analysis | | Multivariate Analysis | |
| --- | --- | --- | --- | --- |
|  | HR (95% CI) | *P* | HR (95% CI) | *P* |
| circPDK1 (low vs. high) | 2.172(1.213-3.890) | 0.009 |  |  |
| Age (≥ 60 vs. < 60) | 1.202(0.692-2.091) | 0.514 |  |  |
| Gender (male vs. female) | 0.831(0.482-1.435) | 0.507 |  |  |
| Pathological stage (I-II vs. III-IV) | 3.411(1.992-5.839) | <0.001 |  |  |
| T stage (T1-2 vs. T3-4) | 2.784(1.619-4.785) | <0.001 |  |  |
| Lymph node metastasis (N0 vs. N1-2) | 3.120(1.654-5.884) | <0.001 |  |  |
| Distant metastasis (M0 vs. M1) | 5.247(2.443-11.268) | <0.001 | 3.631(1.385-9.518) | 0.009 |
